# pH-responsive magnetic Fe_3_O_4_ modified chitosan nanoparticles loaded with β-acids to improve colorectal cancer treatment

**DOI:** 10.1016/j.mtbio.2025.102151

**Published:** 2025-07-29

**Authors:** Songlin Guo, Xia Qiao, Lu Ding, Jiayue Liu, Yanan Xu, Jia Cao, Weidong Tian, Duan Ma, Xu Zhang, Bingren Tian

**Affiliations:** a Surgery Laboratory, Institute of Medical Sciences, General Hospital of Ningxia Medical University, Yinchuan, Ningxia, China; b State Key Laboratory of Quality Research in Chinese Medicine, Institute of Chinese Medical Sciences, University of Macau, Macao, China; c State Key Laboratory of Genetic Engineering and Collaborative Innovation Center for Genetics and Development, School of Life Sciences, Fudan University, Shanghai, China; d Department of Biochemistry and Molecular Biology, Research Center for Birth Defects, Institutes of Biomedical Sciences, Key Laboratory of Metabolism and Molecular Medicine, Ministry of Education, School of Basic Medical Sciences, Fudan University, Shanghai, China

**Keywords:** Chitosan, Hops β-acids, Fe_3_O_4_, pH+magnetic nanoparticle, Antibacterial, Colorectal cancer

## Abstract

Oral drug delivery systems designed for antitumor drug administration in colorectal cancer treatment encounter substantial challenges regarding effective delivery, controlled release, and intestinal microbiota homeostasis. In this study, dual-stimulation (pH + magnetic) responsive chitosan nanoparticles (Fe_3_O_4_/chitosan (CS)/TPP) loaded with hops β-acids were synthesized for colorectal cancer treatment. *In vitro* experiments revealed that the fabricated nanoparticles demonstrated sizes ranging from 233 to 381 nm, with zeta potential values exceeding 10 mV. Release studies indicated that β-acids release was pH-dependent. Notably, the β-acids-loaded nanoparticles proved antimicrobial activity through membrane protein interactions. Cell proliferation assays confirmed that these nanoparticles effectively eliminated HCT116 cells while showing minimal toxicity toward NCM460 cells. Following oral administration to mice with in situ colorectal cancer, histopathological evaluation demonstrated that the nanoparticles induced apoptosis at the tumor site, leading to reduced tumor growth. Additionally, the drug-loaded nanoparticles showed enhanced antitumor efficacy compared to free 5-FU. The findings suggest that these nanoparticles significantly increase beneficial bacterial populations while decreasing levels of harmful bacteria. These dual-function nanoparticles, which exhibit both chemotherapeutic and antimicrobial properties, present a promising novel strategy for treating colorectal cancer.

## Highlights

• It is fabricated pH + magnetic responsive chitosan nanoparticles for CRC therapy.
• Chitosan nanoparticles could be oral drug delivery platforms.
• β-acids could be controlled release form nanoparticles.
• Nanoparticles exhibit well chemotherapy to CRC.

## Graphical abstract

**Figure:**
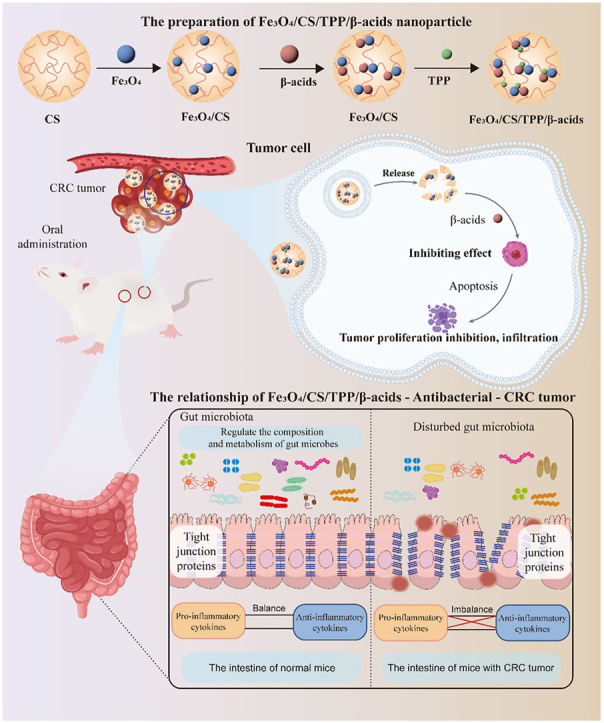

**Statement of significance**

Conventional chemotherapy drugs for colorectal cancer (CRC) treatment exhibit significant side effects that compromise therapeutic efficacy. β-acids, a natural compound extracted from hops, demonstrates both anti-cancer and antibacterial properties, offering potential benefits in CRC clinical treatment. This study presents a multifunctional nanodrug (Fe_3_O_4_/CS/TPP/β-acids), which incorporates β-acids, chitosan, and Fe_3_O_4_ nanoparticles into an integrated delivery system. The nanodrug exhibits enhanced therapeutic efficacy in treating in situ colorectal cancer in mouse models, as evidenced by histopathological analysis, which reveals nanoparticle-induced apoptosis at tumor sites, leading to reduced tumor growth. Additionally, these nanoparticles effectively increase beneficial bacterial populations while reducing pathogenic bacteria. The dual chemotherapeutic and antibacterial properties of these nanoparticles represent an innovative approach to CRC treatment.

## 1 Introduction

Colorectal cancer (CRC), accounting for 9.4 % of global mortality annually, represents a prevalent malignant tumor of the gastrointestinal tract [1]. Current therapeutic approaches primarily involve surgical intervention and chemotherapy. However, patient mortality rates remain elevated due to cancer recurrence and metastasis. While gut microbiota homeostasis maintains equilibrium during cancer treatment, conventional chemotherapeutic agents for CRC management, including 5-fluorouracil, mitomycin, adriamycin, *etc.*, demonstrate significant toxicity, adverse effects, limited selectivity, and insufficient *in vivo* targeting [2,3]. Most of these medications require intravenous administration, with limited options available for oral delivery, such as uracil-tegafur tablets and capecitabine tablets. Although oral drug administration potentially increases patient acceptance and compliance compared to alternative delivery methods, it faces numerous challenges, including poor drug solubility, complex gastrointestinal environments, and non-specific drug distribution [4]. Nanotechnology applications in preparing active ingredient-loaded nanoparticles can effectively address oral delivery limitations by enhancing intestinal barrier penetration, reducing systemic adverse effects, and improving therapeutic efficacy [5]. Oral drug delivery additionally represents the most convenient method for modulating intestinal microbiota [6,7]. Thus, developing innovative drug delivery systems for targeted colonic release of antitumor agents holds significant promise for CRC treatment [8], potentially enhancing drug targeting efficacy while reducing required dosages.

Extended use of chemotherapeutic drugs has led to patient resistance development. Given the lengthy development cycles and substantial research costs associated with new drug development, researchers have increasingly investigated natural products, particularly plant-derived antitumor substances, representing a frontier in antitumor research [9,10]. Hops (*Humulus lupulus*), a globally cultivated perennial plant, produces resin as a significant secondary metabolite containing various bioactive compounds, including α-acids, β-acids, α-sclerotin, β-sclerotin, polyphenols, and volatile oils [11]. β-acids, extracted from hops, demonstrate significant inhibitory effects against CRC cells (Caco-2, SW620, HT29, HCT116), prostate cancer cells (PC3, DU145), and breast cancer cells (MCF-7, MDA-MB-231) [12]. Furthermore, β-acids exhibit broad-spectrum bacterial growth inhibition. The dual anti-cancer and antibacterial properties of β-acids suggest promising potential in the clinical treatment of CRC.

Natural and synthetic biomaterials have been extensively employed in the development of nanocarriers for drug delivery. Notably, natural polymers possess several significant advantages, including low toxicity, biocompatibility, biodegradability, and ready availability. Among various natural biomaterials, chitosan (CS) has garnered considerable attention due to its widespread availability, superior biocompatibility, high mechanical strength, and biodegradability [13]. CS, second only to cellulose in annual global production among polysaccharides, consists of N-acetyl-D-glucosamine and β-(1–4)-D-glucosamine units. Under acidic conditions, CS becomes soluble through protonation, becoming a cationic polymer capable of interacting with various molecules [14]. The hydroxyl and amino groups within its structure enhance its reactivity with other groups. CS-based nanoparticles exhibited broad applicability in drug delivery systems. The diverse materials comprising these nanoparticles significantly influence their physicochemical properties [15,16]. Researchers had successfully encapsulated numerous natural products, including curcumin and doxorubicin, within CS-based nanoparticles [17,18]. *In vitro* studies had demonstrated that these nanoparticles exhibit substantial inhibitory effects on cancer cells, with enhanced anti-cancer efficacy compared to free drugs [19]. For instance, researchers synthesized CS nanoparticles loaded with β-acids by using sodium tripolyphosphate (TPP) as a cross-linking agent [20]. The results indicated that these drug-loaded nanoparticles exhibited significant inhibitory effects on CRC cell lines (HCT116 and HT29), while showing minimal cytotoxicity towards normal colorectal epithelial cells (NCM460). Almeida et al. successfully incorporated camptothecin into polyethylene glycol (PEG) and oleic acid-modified CS micelles. Subsequent *in vivo* anti-cancer studies demonstrated that oral administration of these drug-loaded micelles significantly reduced tumor incidence and inflammatory markers [21]. Similarly, research investigating the use of nanoparticles for paclitaxel delivery in CRC models has confirmed outstanding therapeutic efficacy against CRC and induced changes in both the quantity and composition of the gut microbiota [22]. These results verified that nanoparticles loaded with active ingredients effectively treat CRC and inhibit colorectal tumor progression by modulating the intestinal microbiota. Given CS's superior biocompatibility among natural biomaterials, numerous studies have focused on incorporating active small molecules into CS nanoparticles [23]. This approach has transformed drug release profiles from initial burst release to sustained release, thereby extending the duration of drug action, enhancing bioavailability, and reducing the frequency of administration. The stomach typically maintains a pH of approximately 1.2, while the small intestine ranges from pH 6.0 to 6.8. The terminal small intestine exhibits pH 6.8 to 7.0, and the colonic region varies between pH 6.5 and 7.5. Previous *in vitro* studies on drug release have shown that β-acids release from nanoparticles was influenced by pH variations [20]. The emergence of targeted drug delivery concepts has advanced nanoparticle drug delivery research. By incorporating magnetic molecules into nanoparticles, therapeutic agents can be directed and concentrated at the colorectal site under an applied magnetic field, ultimately inhibiting cancer progression [24,25].

**Scheme 1:**
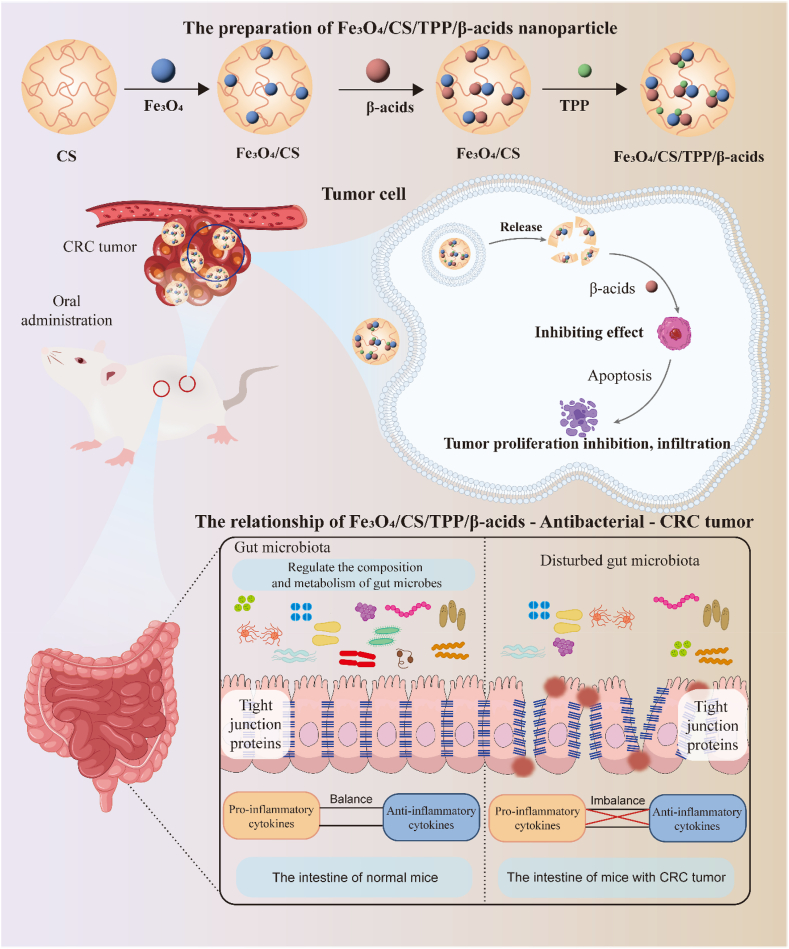
Schematic illustration of combining chemotherapy and antibacterial treatment for CRC by Fe_3_O_4_/CS/TPP/β-acids.

In this study, Fe_3_O_4_-modified CS nanoparticles were synthesized via ionic gelation and subsequently loaded with β-acids, aiming to develop an orally administrable therapeutic agent for the treatment of CT26-induced in situ colorectal cancer and modulation of the intestinal microbiota (Scheme 1). It was hypothesized that the drug-loaded nanoparticles would enhance the gut microbiota and exhibit therapeutic efficacy against colorectal cancer (CRC). We assessed the antitumor activity of nanoparticles in the CT26 colorectal carcinoma model. The outcomes of the *in vivo* antitumor experiments demonstrated that CS nanoparticles loaded with β-acids significantly suppressed both the growth and metastasis of CRC. Concurrently, *in vitro* and *in vivo* action mechanistic studies revealed that the β-acids-loaded nanoparticles inhibited the proliferation of common pathogenic bacteria as well as CRC cells. Building on this, the study employed genome-wide RNA-Seq and 16S rDNA-Seq technologies to explore the potential mechanisms by which β-acids-loaded nanoparticles, both alone and in combination with 5-FU, inhibit CRC from the perspectives of the transcriptome.

This simple, orally active nanoparticle carrier for the targeted delivery of β-acids presents a promising strategy for inhibiting pathogenic microorganism proliferation and achieving sustained efficacy against colorectal cancer (CRC). Additionally, this approach demonstrates significant potential for enhancing the effectiveness of other therapeutic interventions.

## 2 Experimental section

### 2.1 Materials

Chitosan with 90 % deacetylation rate and molecular weight of 750 kDa was obtained from Shanghai Lanji Technology Development Co., Ltd. (Shanghai, China). Hop extracts (supercritical CO_2_ extraction) were supplied by Xinjiang Sapporo Agricultural Technology Development Co., Ltd. Hexane, sodium tripolyphosphate, sodium hydroxide, anhydrous ethanol, disodium hydrogen phosphate, potassium dihydrogen phosphate, sodium chloride, ammonia, and acetic acid were acquired from Shanghai Guangnuo Chemical Technology Co., Ltd. Yeast extract powder was obtained from OXOID (UK). Agar powder was sourced from Coolaber Biotechnology Co., Ltd. 5-Fluorouracil (5-FU), ferric chloride, coumarin 6, and ferrous chloride were purchased from Shanghai Macklin Biotechnology Co., Ltd. All chemical reagents were used as received without further purification.

HCT116 cells (human colorectal cancer cells) and NCM460 (human normal colon epithelial cells) obtained from Shanghai Cell Bank (Shanghai, China) were cultured in McCoy's 5A whole medium (Gibco, USA). The medium contained 10 % (v/v) fetal bovine serum (FBS) (Gibco, USA) and 100 U/mL penicillin-streptomycin (PenStrep) (Gibco, USA). All cells were maintained at 37 °C (5 % CO_2_) in humidified environment. Prior to cell proliferation analysis, each cell line was harvested by pancreatic enzymes (Gibco, USA) and adjusted to the appropriate cell concentration.

### 2.2 Fe_3_O_4_ nanoparticle preparation

FeCl_2_ (1.59 g) and FeCl_3_ (4.32 g) were dissolved in distilled water (40 mL). Subsequently, 12 mL of NH_4_OH was added dropwise, and the mixture was reacted at 50 °C for 2 h. The precipitate was collected by centrifugation and freeze-dried to obtain Fe_3_O_4_ nanoparticles.

### 2.3 Nanoparticle preparation

CS (600 mg) was dissolved in 200 mL of acetic acid solution (1 % v/v), obtained CS solution (3 mg/mL). β-acids were extracted following the method described by Tian et al. [20] and characterized by HPLC (Fig. S1). β-acids (1.8 g) were dissolved in ethanol (40 mL) to prepare a solution (45 mg/mL). Various volumes of β-acids/ethanol solution (7, 14, 22.5 mL) were added dropwise to the CS solution and stirred for 1 h to ensure homogeneity. Fe_3_O_4_ nanoparticles (300 mg) were dispersed in water (20 mL) by using ultrasonication, added dropwise to the reaction solution, and reacted for 1 h. Finally, sodium tripolyphosphate solution (2 mg/mL) was added dropwise while stirring at 800 rpm, 25 °C for 1 h. The nanoparticles were collected by centrifugation at 12000×*g* for 10 min at 4 °C and washed three times with deionized water. The final product was obtained through freeze-drying. Blank nanoparticles were prepared following the same procedure without β-acids (Table S1).

For coumarin 6-loaded nanoparticles, the preparation method remained identical to that of β-acids-loaded nanoparticles, with coumarin-6 is replaced with β-acids.

### 2.4 Characterization

CS, β-acids, TPP, Fe_3_O_4_, Fe_3_O_4_/CS/TPP and 9Fe_3_O_4_/CS/TPP/β-acids powders were compressed with KBr and analyzed at room temperature by Vertex 70 FTIR spectrometer (Bruker, Beijing, China).

D8 Advance diffractometer (Bruker AXS, Germany) was utilized to analyze β-acids (powdered), CS (powdered), TPP (powdered), Fe_3_O_4_ (powdered), Fe_3_O_4_/CS/TPP (powdered) and 9Fe_3_O_4_/CS/TPP/β-acids (powdered). The X-ray energy spectra of the samples were measured under Cu Kα radiation at 40 kV and 40 mA. The scans were conducted in 45 steps of 10 s each in the range of 2θ = 5°–35° at room temperature (25 °C).

The Fe_3_O_4_/CS/TPP, 3Fe_3_O_4_/CS/TPP/β-acids, 6Fe_3_O_4_/CS/TPP/β-acids, and 9Fe_3_O_4_/CS/TPP/β-acids powder samples were examined by SEM (Regulus 8100, Hitachi, Tokyo, Japan) at an excitation voltage of 5.0 kV.

The morphological characteristics of Fe_3_O_4_, Fe_3_O_4_/CS/TPP, 3Fe_3_O_4_/CS/TPP/β-acids, 6Fe_3_O_4_/CS/TPP/β-acids, and 9Fe_3_O_4_/CS/TPP/β-acids powder samples were analyzed by TEM (Tecnai G2 20, FET, USA).

The Zetasizer Nano was employed to measure particle size, polydispersity index and zeta point position analysis of nanoparticles (Fe_3_O_4_/CS/TPP, 3Fe_3_O_4_/CS/TPP/β-acids, 6Fe_3_O_4_/CS/TPP/β-acids and 9Fe_3_O_4_/CS/TPP/β-acids). The samples were homogeneously dispersed in ultrapure water (pH = 7.4) by ultrasound before measurement.

Magnetic properties of Fe_3_O_4_ and 9Fe_3_O_4_/CS/TPP/β-acids nanoparticles were analyzed by using PPMS-9 (VSM) developed by Quantum Design, USA.

A Varian Inova-400 NMR spectrometer was utilized to determine the ^1^HNMR spectra of β-acids (Fig. S2). The solvent dimethyl sulfoxide (DMSO)-*d6* was selected, and the resonance at 2.49 ppm was apparent.

### 2.5 Evaluation of encapsulation efficiency and loading capacity

A quantitative amount of drug-loaded nanoparticles (10 mg) was weighed and centrifuged at 12000 rpm for 10 min at 4 °C, the supernatant was collected and measured by HPLC (Y = 0.0089X + 0.1705, R^2^ = 0.9929, Fig. S3). The encapsulation efficiency (EE) and LC of β-acids in nanoparticles were calculated according to the following equation:

(1) $$EE\left(\%\right)=\frac{Total\;amount\;of\;loaded\;\beta -acids}{Initial\;of\;\beta -acids}\times 100\%$$

(2) $$LC\left(\%\right)=\frac{Total\;amount\;of\;loaded\;\beta -acids}{Weight\;of\;nanoparticle}\times 100\%$$

### 2.6 *In vitro* drug release and model fitting

Accurately weighed 5 mg of different drug-loaded nanoparticles were placed in dialyzed cellulose membrane containing 30 mL of PBS (pH = 1.2, 6.6 and 7.4) and shaken at 37 °C, 220 rpm. At predetermined time intervals (0, 15 min, 30 min, 1 h, 2 h, 4 h, 6 h, 8 h, 10 h, 12 h and 24 h), 2 mL of release medium was extracted to determine the absorbance and 2 mL of fresh buffer solution was added to maintain constant total volume. Based on the absorbance at different time points, drug release data was obtained, and a mathematical model was fitted.

(3) $$\text{First}\;\text{order}\;\;\text{model}:\frac{M_{t}}{M_{\infty }}=a\left(1-e^{k_{1}t}\right)$$

(4) $$\text{Higuchi}\;\;\text{model}:M_{t}=k_{2}t^{0.5}+a$$

(5) $$\text{Zero}\;\text{order}\;\;\text{model}:\frac{M_{t}}{M_{\infty }}=k_{3}t+a$$

(6) $$\text{Ritger}-\text{peppas}\;\;\text{model}:\frac{M_{t}}{M_{\infty }}=k_{2}t^{n}$$

where $\frac{M_{t}}{M_{\infty }}$ is the release percentage of β-acids at time t; k_1_, k_2_, k_3,_ and k_4_ are the rate constant associated with the diffusion process; n is the diffusion index, which represents the parameters related to the release mechanism; a is a constant.

### 2.7 Antibacterial activity experiment

To evaluate the bacteriostatic properties of drug-carrying nanoparticles, three bacterial strains were obtained from the Key Laboratory of Microbiology, General Hospital of Ningxia Medical University: *Escherichia coli ATCC 25922*, *Staphylococcus aureus ATCC 25923*, and *Acinetobacter baumannii ATCC17978*. The bacteria were cultured in LB medium at 37 °C.

The bacterial turbidity was adjusted using 0.9 % NaCl solution to achieve a final concentration of optical density (OD)_600 nm_ = 0.5. β-acids and drug-carrying nanoparticles were dispersed in ethanol to prepare solutions of varying concentrations (8, 4, 2, 1, 0.5, 0.25, 0.125, and 0.0625 mg/mL). A 96-well plate was prepared with 50 μL of bacterial solution (concentration of 1 × 10^7^ CFU/mL) and 100 μL of drug solution at different concentrations (8, 4, 2, 1, 0.5, 0.25, 0.125, 0.0625 mg/mL), followed by incubation at 37 °C for 24 h. Subsequently, 100 μL of the cultured bacterial solution was spread on LB solid medium for 24 h, and bacterial growth was observed and documented.

### 2.8 Molecular docking

The analysis focused on the three primary homologous molecules of β-acids (n-lupulone, colupulone, and adlupulone). Molecular docking simulations were performed using the Hex version 8.0 software package. Selected bacterial surface membrane proteins (*E. coli* PDB IDs: 1QJ8, 7TSZ, 8BO2; *S. aureus* PDB IDs: 4ANN, 4WVJ, 6U0O; *A. baumannii* PDB IDs: 44RL9, 5DL7, 6GL9) served as receptors, with β-acids as ligands. The docking results were visualized with PyMOL 1.7 (Schrödinger, LLC).

### 2.9 Cytotoxicity study by CCK-8 assay

In selecting experimental cell lines, the primary rationale for choosing HCT116 cells is their significant advantages as one of the most extensively utilized human cell lines in colorectal cancer research, particularly for *in vitro* functional studies. HCT116 is frequently cited as a preferred model in numerous *in vitro* antitumor investigations [[26], [27], [28]]. This widespread application is attributable not only to the ease of experimental manipulation but also to the cell line's high biological relevance, well-characterized genetic background, and exceptional experimental reproducibility, all of which contribute to providing reliable data for *in vitro* mechanistic research.

The toxicity assessment of β-acids, blank nanoparticles (Fe_3_O_4_/CS/TPP) and drug-loaded nanoparticles (3Fe_3_O_4_/CS/TPP/β-acids, 6Fe_3_O_4_/CS/TPP/β-acids, and 9Fe_3_O_4_/CS/TPP/β-acids) was conducted by HCT116 and NCM460 cells. The cells were seeded in 96-well plates at a density of 1 × 10^4^ cells per well and allowed to attach for 6 h. After attachment, the medium was replaced with a drug-containing medium and incubated for 24 h. Subsequently, the drug-containing medium was replaced with fresh medium, and 10 μL of CCK-8 solution was added to each well. Following 1 h incubation at 37 °C, the OD was measured at 450 nm. 5-Fluorouracil (5-FU) served as positive control.

### 2.10 Cell migration assay

HCT116 cells (3 × 10^5^ cells/well) were seeded into 6-well culture plates and maintained in saturated humidity with 5 % CO_2_ at 37 °C for 24 h. After complete cell adherence, straight-line scratches were created using 10 μL lance tips, and initial photographs were taken under a microscope as 0 h control. The medium was replaced with a serum-free medium, and cells were incubated at 37 °C with 5 % CO_2_. After 24 h, cells were photographed under microscope, and the data were analyzed by ImageJ software.

### 2.11 High-content live cell imaging

HCT-116 cells were cultured at 37 °C in 5 % CO_2_ incubator until adherence. The medium was removed and replaced with 200 μL of pre-configured drug medium per well. Following 24 h incubation, cells were stained with Hochest 33342 (blue) and Caspase3/7 (green) live cell fluorescent dyes. Images were captured by High-content Live Cell Workstation with 20x lens, and results were analyzed with the workstation's built-in analysis system.

### 2.12 Cellular uptake

HCT116 cells in the logarithmic growth phase were adjusted to 10,000 cells/100 μL and seeded into laser confocal 96-well plates at 100 μL per well (three replicates per group). Fe_3_O_4_/CS/TPP/Coumarin-6 and Fe_3_O_4_/CS/TPP/Coumarin-6+β-acids nanoparticles were added to respective wells. Cells were collected at 0 min, 30 min, 60 min, and 120 min of incubation and stained with Hochest 33342 (blue) live cell fluorescent dye. Observations and images were obtained by laser confocal microscope with 40x objective lens after adjusting the field and focal length.

### 2.13 *In vivo* biosafety

Male C57BL/6J mice, aged 6–8 weeks, were obtained from Wuhan Sevier Biotechnology Co. and maintained in a specific pathogen free (SPF) facility. All animal procedures adhered to the National Research Council's Guide for the Care and Use of Laboratory Animals (8th edition, NIH Publication, 2011). The experimental procedures complied with the regulations of the Medical Ethics Committee of General Hospital of Ningxia Medical University (KYLL-2022-1009 and KYLL-2024-1031).

To assess the biocompatibility of control, β-acids, 5-FU, Fe_3_O_4_/CS/TPP, 9Fe_3_O_4_/CS/TPP/β-acids and 9Fe_3_O_4_/CS/TPP/β-acids+5-FU groups (n = 3 in each group), nanoparticles were administered orally to 6-8-week-old male C57BL/6J mice. The mice received 200 mg/kg every 2 days (β-acids, 5-FU, Fe_3_O_4_/CS/TPP, and 9Fe_3_O_4_/CS/TPP/β-acids), while 5-FU was administered via injection (25 mg/kg). After two weeks, the mice were euthanized, and major organs including heart, liver, spleen, lung and kidney were subjected to H&E staining.

### 2.14 *In vivo* tumor model therapeutic efficiency

The selection of CT26 cells is primarily intended to fulfill the construction requirements of the *in vivo* model. This cell line, originating from BALB/c mice, can be directly inoculated into syngeneic mice, thereby facilitating efficient tumor formation. The homogeneous transplanted colorectal cancer mouse model developed from this cell line exhibits characteristics such as rapid tumor growth, high stability, and minimal inter-group variability. This model more accurately simulates the *in vivo* tumor growth process while fully preserving the antitumor immune response. Consequently, it serves as an ideal platform for the *in vivo* evaluation of drug efficacy. Based on these attributes, we have chosen the CT26 allograft colorectal cancer mouse model for subsequent *in vivo* drug efficacy evaluation experiments.

CT26 cells (5 × 10^5^ cells/each, diluted with 25 μL PBS) were injected into the cecum wall of 6∼8-week-old C57BL/6J mice to establish in situ colorectal cancer model. All animal procedures adhered to the National Research Council's Guide for the Care and Use of Laboratory Animals (8th edition, NIH Publication, 2011). The experimental procedures complied with the regulations of the Medical Ethics Committee of General Hospital of Ningxia Medical University (KYLL-2022-1009 and KYLL-2024-1031). In situ colorectal cancer growth was monitored weekly using a Bruker In Vivo MS FX PRO imager. The model mice were divided randomly into control, 5-FU, β-acids, Fe_3_O_4_/CS/TPP, 9Fe_3_O_4_/CS/TPP/β-acids and 9Fe_3_O_4_/CS/TPP/β-acids+5-FU groups (n = 5 in each group). Drug administration occurred via gavage (200 mg/kg) every two days for two weeks, with drug-carrying nanoparticles concentrated at the colorectal site through magnetic field control. 5-FU served as a positive control drug, administered through intraperitoneal injection (25 mg/kg) weekly. After euthanization, colorectal tissue, tumor tissue, and feces were collected for H&E staining or RNA-Seq analysis.

### 2.15 Statistical analysis

Statistical analysis employed SPSS 17.0 statistical analysis software, utilizing the independent Student's T test for two-group comparisons and one-way ANOVA for multiple groups. Normality and variance homogeneity tests were performed on all data utilized in the manuscript. Data are presented as mean ± SD. *P* values of <0.05 indicated statistical significance. Error bars represent the estimated measurement error or value uncertainty.

## 3 Results and discussion

### 3.1 Physical properties and characterization of nanoparticles

**Fig. 1:**
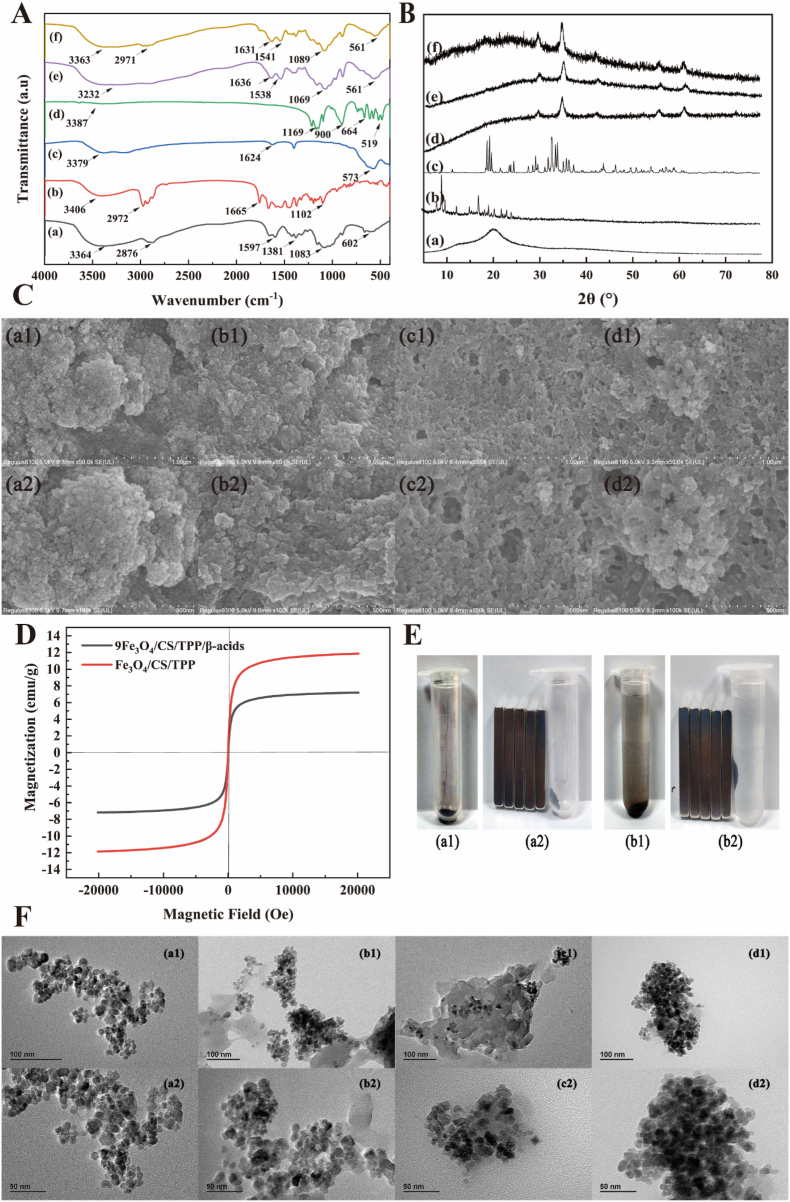
(A) FTIR spectra of (a) CS, (b) β-acids, (c) Fe_3_O_4_, (d) TPP, (e) Fe_3_O_4_/CS/TPP and (f) 9Fe_3_O_4_/CS/TPP/β-acids; (B) XRD patterns of (a) CS, (b) β-acids, (c) TPP, (d) Fe_3_O_4_, (e) Fe_3_O_4_/CS/TPP and (f) 9Fe_3_O_4_/CS/TPP/β-acids; (C) SEM images of Fe_3_O_4_/CS/TPP: (a1) 1 μm, (a2) 500 nm; 3Fe_3_O_4_/CS/TPP/β-acids: (b1) 1 μm, (b2) 500 nm; 6Fe_3_O_4_/CS/TPP/β-acids: (c1) 1 μm, (c2) 500 nm; and 9Fe_3_O_4_/CS/TPP/β-acids: (d1) 1 μm, (d2) 500 nm; (D) Hysteresis loops of Fe_3_O_4_/CS/TPP and Fe_3_O_4_/CS/TPP/β-acids measured at 300K; (E) External magnetic field response of 9Fe_3_O_4_/CS/TPP/β-acids (a1 and a2) in dry conditions, (b1 and b2) in aqueous solution; (F) TEM images of Fe_3_O_4_/CS/TPP: (a1) 100 nm, (a2) 50 nm; 3Fe_3_O_4_/CS/TPP/β-acids: (b1) 100 nm, (b2) 50 nm; 6Fe_3_O_4_/CS/TPP/β-acids: (c1) 100 nm, (c2) 50 nm; and 9Fe_3_O_4_/CS/TPP/β-acids: (d1) 100 nm, (d2) 50 nm.

In this study, the synthesized nanoparticles were initially characterized by FTIR. CS (Fig. 1A(a)) exhibited an absorption peak at 3364 cm^−1^, which previous studies have attributed to the -OH vibration [29]. The absorption peaks observed at 2876 cm^−1^ correspond to asymmetric and symmetric vibrations of C-H. The peaks near 1655 cm^−1^, 1597 cm^−1^, and 1381 cm^−1^ correspond to different vibrational modes of N-H bonds (amide I, amide II, and amide III) in CS. The peak at 1083 cm^−1^ relates to C-O stretching vibration, consistent with previously published findings. β-acids, the active component, displayed a strong, broad -OH absorption peak at 3406 cm^−1^ in FTIR characterization (Fig. 1A(b)). Additionally, the absorption peak near 2970 cm^−1^ represented C-H stretching vibration absorption. The peak at 1665 cm^−1^ was identified as C=O absorption characteristic of β-acids, aligning with literature findings [20]. Due to the magnetic Fe_3_O_4_ component, a strong peak at 573 cm^−1^ and a weak peak at 1624 cm^−1^ emerged from Fe-O bond stretching vibration (Fig. 1A(c)). TPP, serving as a cross-linking agent, showed absorption spectra at 1169 cm^−1^ and 900 cm^−1^, corresponding to P-O and P-O-P stretching vibrations, respectively (Fig. 1A(d)). Following nanoparticle formation through ionic gelation, studies have documented that intermolecular -OH interactions can cause peak shifts [30]. This phenomenon was observed in the present experiment. Fe_3_O_4_/CS/TPP exhibited a broad peak at 3232 cm^−1^, potentially attributable to intermolecular hydrogen bonding (Fig. 1A(e)). The CS amide bonding peaks shifted to 1636 cm^−1^, 1538 cm^−1^, and 1395 cm^−1^. The Fe_3_O_4_ peak at 1624 cm^−1^ shifted to 1636 cm^−1^. These amide bonding changes indicate electrostatic interactions between the CS, TPP and Fe_3_O_4_, forming a complex structure. Upon β-acids addition, FTIR spectra showed further changes. The -OH of β-acids interaction with blank nanoparticles (Fe_3_O_4_/CS/TPP) shifted the -OH absorption peak to 3363 cm^−1^ and generated a β-acids-specific peak at 2971 cm^−1^. Compared to blank nanoparticles, peaks at 1636 cm^−1^, 1538 cm^−1^, and 1395 cm^−1^ shifted to 1631 cm^−1^, 1541 cm^−1^, and 1390 cm^−1^ (Fig. 1A(f)). These FTIR results confirmed the successful preparation of stimulus-responsive nanoparticles loaded with β-acids.

The XRD spectra of CS, β-acids, TPP, Fe_3_O_4_, Fe_3_O_4_/CS/TPP, and 9Fe_3_O_4_/CS/TPP/β-acids are shown in Fig. 1B. CS exhibited high-intensity diffraction peak at 2θ = 20.25°, which indicated that amino and hydroxyl groups in CS formed hydrogen bonds and demonstrated strong crystallinity. β-acids displayed characteristic absorption peaks at 2θ = 8.31°, 9.40°, 10.20°, 12.71°, 15.62°, 17.60°, 19.84°, 21.13°, 22.67°, 23.79°, and 24.83°, suggesting a certain degree of crystallinity. The crosslinker TPP exhibited absorption peaks at 2θ = 11.31°, 19.65°, 25.04°, 29.89°, 33.57°, 45.05°, 55.59° and 58.96°. XRD characterization of pure Fe_3_O_4_ revealed peaks at 2θ = 30.44°, 35.79°, 43.21°, 53.29°, 57.01°, and 62.99°, corresponding to the XRD standard card (JCPDS card NO.19-629). The analysis confirmed that Fe_3_O_4_ synthesis comprises (220), (311), (400), (422), (511), and (620) crystal planes. The grain size of pure Fe_3_O_4_, calculated using Scherrer's equation, was approximately 12.91 nm, potentially contributing to the system's hypermagnetism. Following nanoparticle formation with CS, TPP, and Fe_3_O_4_, the diffraction peaks shifted and broadened. Previous studies attributed this phenomenon to the formation of intermolecular hydrogen bonds between TPP and CS, resulting in CS losing its natural crystal structure [31]. Additionally, the diffraction peak width correlates with crystal size and amorphous phase presence. The originally ordered CS molecular arrangement was disrupted during nanoparticle formation, while Fe_3_O_4_ maintained its supermagnetic properties. β-acids loading resulted in broader diffraction peaks, attributed to the introduction of β-acids and the formation of an amorphous structure. Bulatao et al. noted that lutein could modify the crystalline form of polymers without affecting Fe_3_O_4_'s crystalline nature [32]. Similar findings were reported by research teams by celecoxib, clove essential oil, and rapamycin as active ingredients in CS nanoparticles [[33], [34], [35]].

Fig. 1C presents typical SEM images of blank (Fe_3_O_4_/CS/TPP) and drug-loaded nanoparticles (9Fe_3_O_4_/CS/TPP/β-acids). As shown in Fig. 1C(a), Fe_3_O_4_ nanoparticles combined with CS and TPP demonstrate a well-spherical structure with a rough surface. The nanoparticle size increased proportionally after β-acids loading. These SEM images provide substantial evidence for the successful synthesis of dual-stimulation-responsive nanoparticles containing β-acids. The composite nanoparticles tend to form stable agglomerates due to their small particle size, high surface energy, and surface activity [36]. Ding et al. observed that nanoparticle size increased with higher 5-FU content [37]. It should be noted that SEM analysis, conducted under dry conditions, may not accurately represent the particles' state in liquid form.

To evaluate the magnetic properties of the nanoparticles, magnetization hysteresis loops of unloaded and drug-loaded Fe_3_O_4_ magnetic nanoparticles were measured at 300 K (Fig. 1D). The results revealed that the saturation magnetization strength (σs) of the blank magnetic nanoparticles was approximately 11.87 emu/g, while that of the drug-loaded magnetic nanoparticles was approximately 7.19 emu/g. Fig. 1D and Fig. S4 demonstrated that the σs decreased after coating β-acids. This reduction in magnetization strength primarily results from the polymer coating on the magnetic nanoparticles' surface. During experiments, the magnetic nanoparticles were successfully immobilized on a test tube wall using a magnetic field for 60 s (Fig. 1E). Macroscopic experiments confirmed that the magnetic nanoparticles modified with CS, TPP, and β-acids maintained sufficient saturation magnetization strength, enabling facile solid-liquid separation (Fig. 1E). Consistent with these findings, magnetic microspheres produced through copolymerization exhibited notably lower saturation magnetization strength [38]. The decreased saturation magnetization intensity of Fe_3_O_4_/CS/TPP and 9Fe_3_O_4_/CS/TPP/β-acids compared to Fe_3_O_4_ likely results from the formation of magnetically dead layers of CS, TPP, and β-acids. Surface crystalline disorder may also substantially reduce the nanoparticles' saturation magnetization intensity due to the significant proportion of surface atoms [39]. Furthermore, Fig. 1D showed the hysteresis loop exhibited no residual at low magnetic fields, indicating negligible coercivity without an applied magnetic field. This observation suggested both Fe_3_O_4_/CS/TPP and 9Fe_3_O_4_/CS/TPP/β-acids display superparamagnetic properties at room temperature, a crucial characteristic for pharmaceutical biomedical applications.

**Fig. 2:**
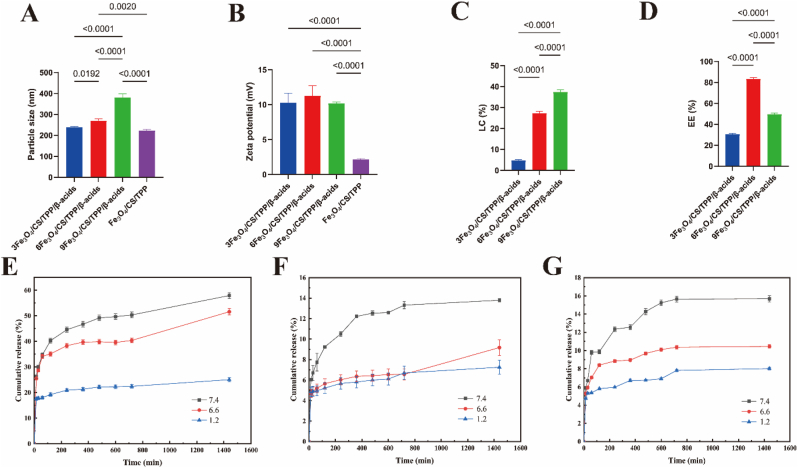
Physical properties of β-acids loaded Fe_3_O_4_/CS/TPP nanoparticle. (A) Particle size, (B) zeta potential, (C) LC, (D)EE. *In vitro* release profile of β-acids from nanoparticles. (E) 3Fe_3_O_4_/CS/TPP/β-acids, (F) 6Fe_3_O_4_/CS/TPP/β-acids, and (G) 9Fe_3_O_4_/CS/TPP/β-acids.

The morphological characteristics of Fe_3_O_4_, Fe_3_O_4_/CS/TPP, 3Fe_3_O_4_/CS/TPP/β-acids, 6Fe_3_O_4_/CS/TPP/β-acids and 9Fe_3_O_4_/CS/TPP/β-acids nanoparticles were examined using TEM (Fig. 1F). The synthesized magnetic Fe_3_O_4_ exhibited predominantly ellipsoidal morphology (Fig. S5). Fe_3_O_4_/CS/TPP nanoparticles demonstrated an average particle size of 233 nm (Fig. 2A). The drug-carrying nanoparticles displayed sizes of 238, 270, and 381 nm, respectively, confirming β-acids additions significantly influence particle size. The interaction between Fe_3_O_4_ and CS proved challenging to observe under TEM. Drug-loaded nanoparticles exhibited distinct dispersion behavior and aggregated morphology compared to β-acids-free nanoparticles. β-acids also influenced zeta potential of the nanoparticles. Fe_3_O_4_/CS/TPP nanoparticles exhibited zeta potential of +2.16 mV, likely attributable to positively charged amino groups (NH_3_^+^) in CS under acidic conditions, with charge repulsion contributing to the positive potential of the nanoparticles (Fig. 2B). β-acids loaded resulted in significant increases in both range and magnitude of nanoparticle's zeta potential compared to the blank nanoparticles. The elevated zeta potential values correlate with expanded particle size distribution following β-acids addition. These observations align with previous research showing size variations between drug-carrying and blank nanoparticles [40]. Given that bacteria typically carry substantial negative charge, the nanoparticles developed in this study can effectively interact with bacteria, which could lead to bacterial death. Research indicated that highly positive charged nanomaterials enhance bacterial interaction through electrostatic attraction [41].

### 3.2 In vitro release and model fitting

HPLC-UV spectroscopy was utilized to determine the EE and LC of β-acids in nanoparticles (Fig. 2C and D). HPLC-UV analysis revealed that LC ranged from 4.55 to 37.48 %, while the EE ranged from 30.91 to 83.56 %. The drug loading efficiency of the active ingredient was influenced by the interaction between the hydrophobicity of β-acids and the nanocarriers. Furthermore, the continuous increase of LC suggested that the nanoparticles had not reached their maximum loading capacity for β-acids. Additionally, the molecules comprising the nanoparticles formed numerous stable hydrogen bonds between different structural groups. During the final preparation stage, multiple washes with distilled water reduced the β-acids loading on the nanoparticle's outer surface. Previous research indicated that both preparation methods and reagent input ratios influence the loading of active ingredients onto nanoparticles [42].

The primary release mechanism of β-acids from nanoparticles occurs through drug diffusion from the particles into the surrounding medium. Most plant-derived active ingredients exhibit a biphasic release pattern characterized by initial burst release followed by slow release. To evaluate the release characteristics of drug-loaded nanoparticles, they were exposed to simulated body fluid environments (37 °C, pH = 7.4, 6.6, 1.2). The stomach typically maintains a pH near 1.2, while the small intestine ranges from pH 6.0–6.8, with its terminal portion at pH 6.8–7.0, and the colon exhibits pH 6.5–7.5. Results demonstrated that β-acids underwent rapid initial release under various simulated conditions, followed by a decreased release rate over time.

As shown in Fig. 2E, F, G, the drug exhibited rapid release during the first 6 h, likely attributed to β-acids attachment to the nanocarrier surface. During this period (pH = 7.4), approximately 45 %, 11 % and 12 % were released from 3Fe_3_O_4_/CS/TPP/β-acids, 6Fe_3_O_4_/CS/TPP/β-acids and 9Fe_3_O_4_/CS/TPP/β-acids nanoparticles, respectively. Subsequently, the drug diffused gradually from the polymer-based nanocarriers. After 12 h, the release rate decreased markedly due to diminishing drug concentration in the carriers. During the final 12 h, the incremental β-acids release from different drug-carrying nanoparticles decreased substantially. These findings demonstrated that the prepared nanoparticles exhibited time-dependent behavior and effectively prevented excessive β-acids release. Furthermore, drug release varied under different pH conditions, with the surface-synthesized nanocomposites displaying pH-responsive characteristics. β-acids release was more pronounced under neutral conditions. The reduced diffusivity under acidic conditions may be attributed to chitosan amino group protonation. The experimental results indicated enhanced stability of β-acids-loaded nanoparticles in acidic conditions. In weakly alkaline buffer solutions, β-acids diffusion increased significantly, potentially due to chitosan chain amine group deprotonation, weakening ionic interactions between cationic chitosan and anionic TPP, thereby accelerating β-acids release. Literature suggested that during initial drug release (burst release), water interacts with chitosan surface hydroxyl and amino groups, leading to particle hydration [43]. In the subsequent phase (slow release), solute molecules penetrate the nanoparticles, creating osmotic pressure differences that facilitate gradual β-acids release. These distinct release processes correlate closely with the substance and active ingredient properties. Additionally, reduced β-acids release under acidic conditions explains gastric juice's ability to minimize release from nanoparticles, while positively charged nanoparticles remain stable. Conversely, β-acids release increases substantially under neutral conditions, attributed to CS deprotonation. The ionization of protonated amine groups enhances CS nanoparticle swelling, resulting in slow active ingredient diffusion in acidic media. To better analyze the β-acids release mechanism, various kinetic models were applied (Table S2). Analysis revealed the Ritger-peppas model as most appropriate.

Furthermore, β-acids release simulation was studied along the digestion timeline (0–2 h: Simulated gastric fluid at pH = 1.2; 2–8 h: Simulated small intestine at pH = 6.6; 8–24 h: Simulated intestinal fluid at pH = 7.4) (Figs. S6 and S7). In larger volumes of simulated gastric medium, approximately 8–9 % release occurred within 2 h. At higher pH, CS insolubility decelerated drug release, with 19–22 % β-acids release observed at pH = 6.8 over 6 h in the intestines. Upon reaching the small intestinal site, the drug demonstrated sustained release, achieving 37–42 % final release. Additional experiments with digestive enzymes in simulated body fluids revealed that different digestive enzymes could inhibit the release of drugs in drug-loaded nanoparticles (Fig. S7). Reduced direct drug exposure to enterocytes potentially mitigates drug side effects. However, *in vivo* release may differ in the colon due to colonic bacterial enzymes promoting CS degradation compared to *in vitro* conditions. In various PBS buffer solutions, continuous nanoparticle structure erosion by water molecules and drug dissolution may increase nanoparticle size while potentially decreasing particle strength (Fig. S8).

### 3.3 *In vitro* antibacterial and molecular docking

**Fig. 3:**
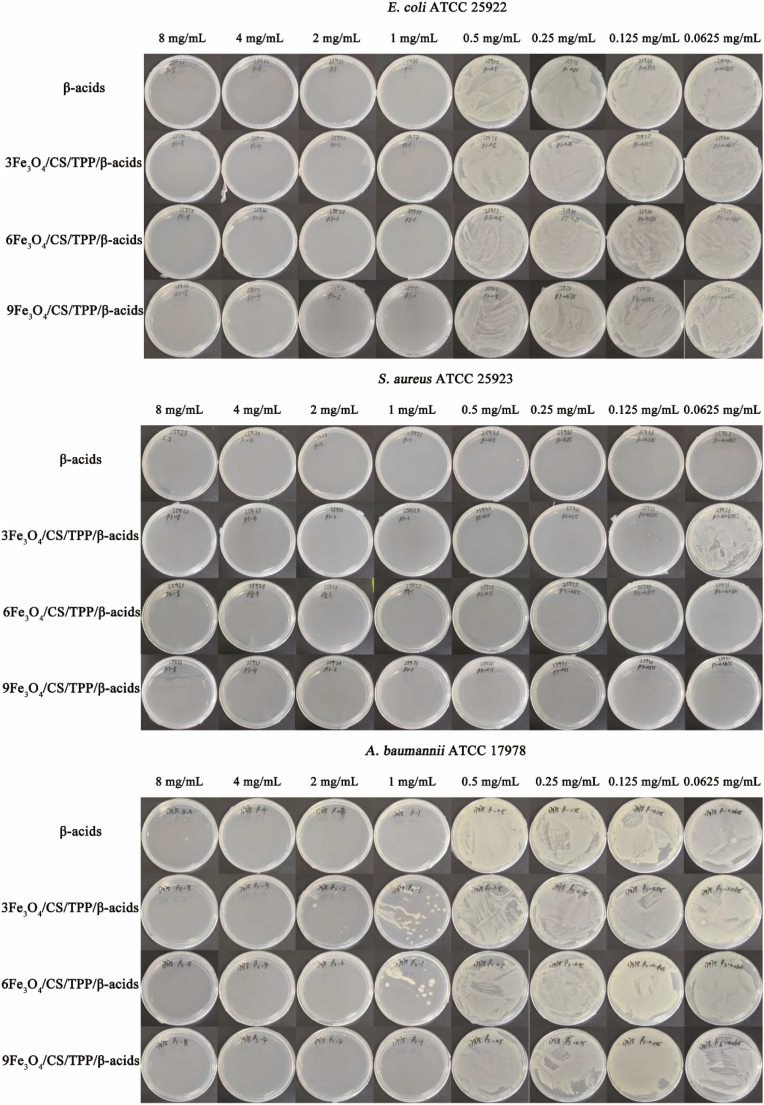
Total number of bacterial colonies of β-acids loaded nanoparticle against bacteria (*E. coli*; *S. aureus*; *A. baumanii*).

The bacterial counting assay evaluated the bacterial inhibitory activity of β-acids and drug-loaded nanoparticles against common clinical bacterial strains (*E. coli*, *S. aureus* and *A. baumannii*) (Fig. 3, Fig. S9 and Table S3). The antibacterial efficacy of β-acids and drug-loaded nanoparticles demonstrated superior performance against *S. aureus* compared to the other two bacteria. Drug-loaded nanoparticles at concentrations exceeding 0.125 mg/mL effectively inhibited *S. aureus*. For Gram-negative bacteria, inhibitory effects required drug-loaded nanoparticle concentrations above 1 mg/mL. The MIC of 3Fe_3_O_4_/CS/TPP/β-acids, 6Fe_3_O_4_/CS/TPP/β-acids and 9Fe_3_O_4_/CS/TPP/β-acids for *E. coli* was 0.25, 0.25, and 0.25 mg/mL, respectively. For *S. aureus*, the MIC was 0.0625, 0.0625 and 0.0625 mg/mL, respectively. For *A. baumannii*, the MIC was 0.25, 0.5 and 0.25 mg/mL, respectively. Molecular docking studies have garnered significant research interest over decades due to their capacity to provide unique insights into biological function mechanisms [44]. This study analyzed β-acids' binding ability to three bacterial surface membrane proteins (Table S4). Fig. S10 revealed β-acids' interaction with surface membrane proteins of *E. coli*, demonstrating hydrogen-bonding interactions with SER-42, ARG-421, SER-732, ARG-583, LEU-124, ALA-123, PHE-127, and GLU-149, with binding energies reaching 6.7 kcal/mol. Fig. S10 illustrated β-acids' interaction with *S. aureus* surface membrane proteins, showing hydrogen bonding interactions with GLU-149, TYR-182, GLU-50, ASN-156, GLU-159, PHE-166, ASP-47, and ASN-168, with binding energies reaching 7.0 kcal/mol. Fig. S10 documented β-acids' interactions with *A. baumannii* surface membrane proteins, exhibiting hydrogen-bonding interactions with GLY-168, ARG-157, ARG-75, ARG-125, ARG-123, ARG-19, ALA-219, SER-217, and PHE-218 with binding energies up to 6.9 kcal/mol.

Disease states significantly alter the gut microbiota structure, and the oral administration of certain drugs can improve the intestinal microenvironment [45]. This experiment utilized pure β-acids as control, demonstrating marked antibacterial activity. β-acids, derived from hop soft resin, exhibit favorable fat solubility. Previous research indicated that β-acids inhibit bacterial growth by disrupting the normal structure, and molecular docking analyses revealed their effects on cell walls and fatty acid biosynthesis pathways [46]. The unique structure of β-acids enhances its hydrophobicity, facilitating bacterial apoptosis. Regarding magnetic nanoparticle interactions with bacteria, studies suggest that bacterial apoptosis occurs through contact between cations (Fe^3+^) and the negatively charged cell membranes, leading to cell death [47,48]. Additionally, evidence suggested that electrostatic interactions between the negative charged of microbial cell walls and the protonated amino groups of CS may alter the cellular pore structure, resulting in antimicrobial effects [49].

Many studies have demonstrated that *E. coli* can promote the progress of colorectal cancer. Jian et al. reported that *E. coli* can promote the carcinogenesis of CRC by inducing DNA alkylation [50]; Mirzarazi et al. found that OmpA protein was overexpressed in the symbiotic *E. coli* B2 phylogenetic group isolated from colorectal cancer patients. This protein significantly reduced the expression of the pro-apoptotic genes Bax and Bak and P53 in the HCT116 cell line [51]. In addition, studies by Lamaudière’ group, and Dehbashi's group showed that *S. aureus* and *A. baumannii* can indirectly affect the CRC process through chronic inflammation or regulation of intestinal microbiota [52,53]. The mice we selected were grown in a sterile environment and did not exhibit three bacteria *in vitro*; however, they are closely related to colorectal cancer. Therefore, *in vitro* research can provide a basis for future colorectal cancer treatment.

### 3.4 *In vitro* cytotoxicity and cellular uptake

**Fig. 4:**
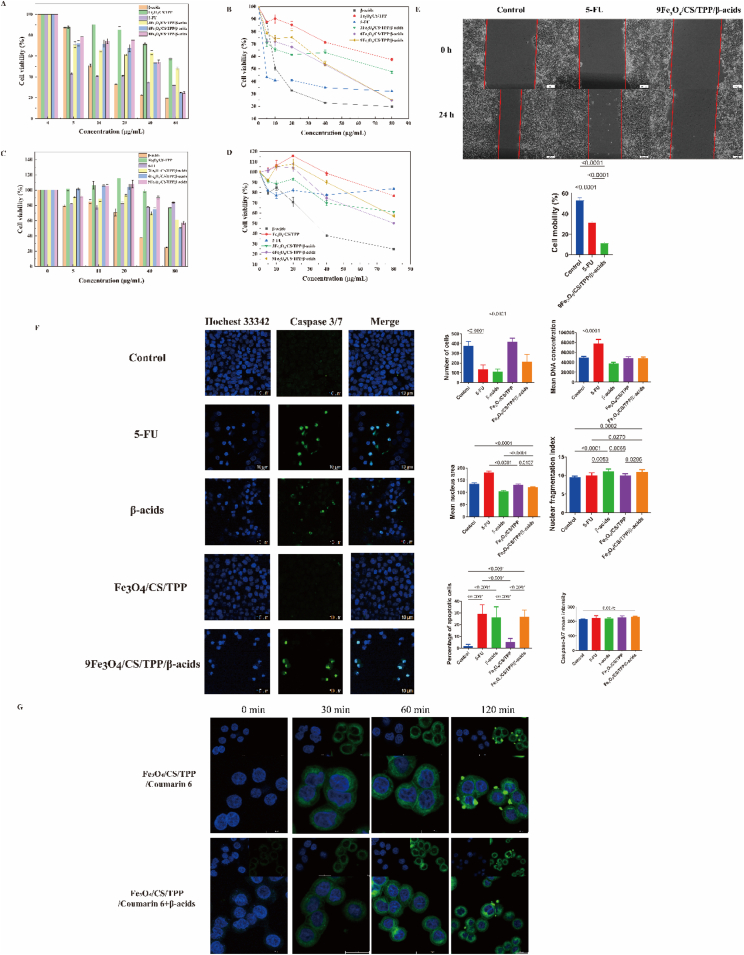
(A and B) of HCT116 (C and D) of NCM460. Cell viability assessment of β-acids, 5-FU, Fe_3_O_4_/CS/TPP, 3Fe_3_O_4_/CS/TPP/β-acids, 6Fe_3_O_4_/CS/TPP/β-acids and 9Fe_3_O_4_/CS/TPP/β-acids on colorectal cancer cells; (E) Cell migration analysis of control, 5-FU and 9Fe_3_O_4_/CS/TPP/β-acids on HCT116; (F) Fluorescence imaging and quantitative analyses of HCT116. Cell nuclei and apoptosis were visualized with hochest 33342 (blue) and caspase-3 marker (green), respectively. Measurement parameters: cell count, mean DNA concentration, mean nucleus area, nuclear fragmentation index, percentage of apoptotic cells and caspase-3/7 mean intensity; (G) Representative CLSM images of HCT116 cells following incubation with Fe_3_O_4_/CS/TPP/coumarin 6 and Fe_3_O_4_/CS/TPP/coumarin 6+β-acids for 6 h. Cell nuclei were labeled with DAPI (blue), and the Fe_3_O_4_/CS/TPP were labeled with coumarin 6 (green).

*In vitro* cytotoxicity of pure β-acids, 5-FU, Fe_3_O_4_/CS/TPP, 3Fe_3_O_4_/CS/TPP/β-acids, 6Fe_3_O_4_/CS/TPP/β-acids and 9Fe_3_O_4_/CS/TPP/β-acids nanoparticles was evaluated by CCK-8 assay against HCT116, CT26 (Fig. S11) and NCM460 cell lines. Fig. 4A and B demonstrate the effect of these six materials on HCT116 cell activity, which decreased as drug concentration increased. At concentrations ranging from 5 to 80 μg/mL, the cell viability for β-acids, 5-FU, Fe_3_O_4_/CS/TPP, 3Fe_3_O_4_/CS/TPP/β-acids, 6Fe_3_O_4_/CS/TPP/β-acids, and 9Fe_3_O_4_/CS/TPP/β-acids was 87 %–19 %, 43 %–31 %, 87 %–57 %, 70 %–47 %, 72 %–24 %, and 78 %–24 %, respectively. Notably, blank nanoparticles in the 5–40 μg/mL range enhanced NCM460 growth (Fig. 4C and D). Additionally, β-acids at high concentrations (40, 80 μg/mL) showed significant inhibitory effects on NCM460 (cell viability below 50 %). The IC_50_ values of HCT116 were 13.25, 1.29, 120.20, 101.70, 33.95, and 38.81 μg/mL for β-acids, 5-FU, Fe_3_O_4_/CS/TPP, 3Fe_3_O_4_/CS/TPP/β-acids, 6Fe_3_O_4_/CS/TPP/β-acids, and 9Fe_3_O_4_/CS/TPP/β-acids, respectively. The IC_50_ values against NCM460 were 32.09, 99.18, 128.10, 77.01, and 87.06 μg/mL for β-acids, Fe_3_O_4_/CS/TPP, 3Fe_3_O_4_/CS/TPP/β-acids, 6Fe_3_O_4_/CS/TPP/β-acids, and 9Fe_3_O_4_/CS/TPP/β-acids. The selectivity index (SI=IC_50_/MIC) of nanoparticles based on cytotoxicity (IC_50_) and MIC serves as crucial indicator for assessing nanoparticle safety (Table S5). The study revealed that 6Fe_3_O_4_/CS/TPP/β-acids nanoparticles demonstrated higher selectivity index compared to the other two nanoparticle groups. The nanoparticles exhibited well selectivity for *S. aureus*.

The concentrations required for antibacterial activity (in the milligram range) appear to be significantly higher than those necessary for anticancer activity (in the microgram range). We thought that this discrepancy in dosage efficacy between antibacterial and antitumor activities may be attributed to two primary factors. Firstly, bacteria possess cell walls and more complex structural features, which confer strong resistance to drug penetration, necessitating higher drug concentrations to effectively penetrate and eradicate bacterial cells. In contrast, tumor cells, being mammalian in nature, exhibit greater membrane permeability to drugs, allowing lower doses to achieve antitumor effects. Secondly, tumor cells undergo rapid division and are highly sensitive to DNA damage, making them susceptible to apoptosis induced by low-dose DNA-damaging agents. Conversely, bacteria exhibit robust stress survival mechanisms, including drug-resistant pumps and multiple repair systems, thereby requiring higher doses for complete eradication.

Furthermore, we evaluated the impact of different treatments (selecting IC_50_ as the experimental concentration) on HCT116 cell migration through a scratch assay, which demonstrated that the drug-loaded nanoparticles effectively inhibited HCT116 cell proliferation (Fig. 4E). Following the treatment of HCT116 cells with 5-FU and drug-loaded nanoparticles, the cell migration rates were observed at 31.71 % and 11.62 %, respectively (Fig. 4E). These findings corroborated the CCK-8 experimental results. The cytotoxic effect of β-acids-loaded magnetic nanoparticles on CRC cells was conclusively demonstrated in HCT116 cells. High-content screening and flow cytometry analysis of drug-loaded nanoparticles' inhibitory effect on HCT116 cells revealed that 9Fe_3_O_4_/CS/TPP nanoparticles significantly suppressed HCT116 cell proliferation and enhanced apoptosis (Fig. 4F and Fig. S12). To assess Fe_3_O_4_/CS/TPP nanoparticle targeting capabilities, nanoparticles were prepared with coumarin 6 alone and in combination with β-acids. The interaction with HCT116 cells was monitored and documented using laser scanning confocal microscopy. As illustrated in Fig. 4G, HCT116 cells showed increasing uptake of nanoparticles loaded with both β-acids and coumarin 6 (green fluorescence) over time. The cytoplasmic fluorescence intensity strengthened progressively with increased incubation duration, indicating time-dependent Fe_3_O_4_/CS/TPP nanoparticle uptake. The proposed mechanism likely involves lectin receptor-mediated cellular internalization, with nanoparticles creating a positive charge aggregation environment [54].

### 3.5 *In vivo* biosafety, anti-cancer therapy and intestinal microorganisms regulation

**Fig. 5:**
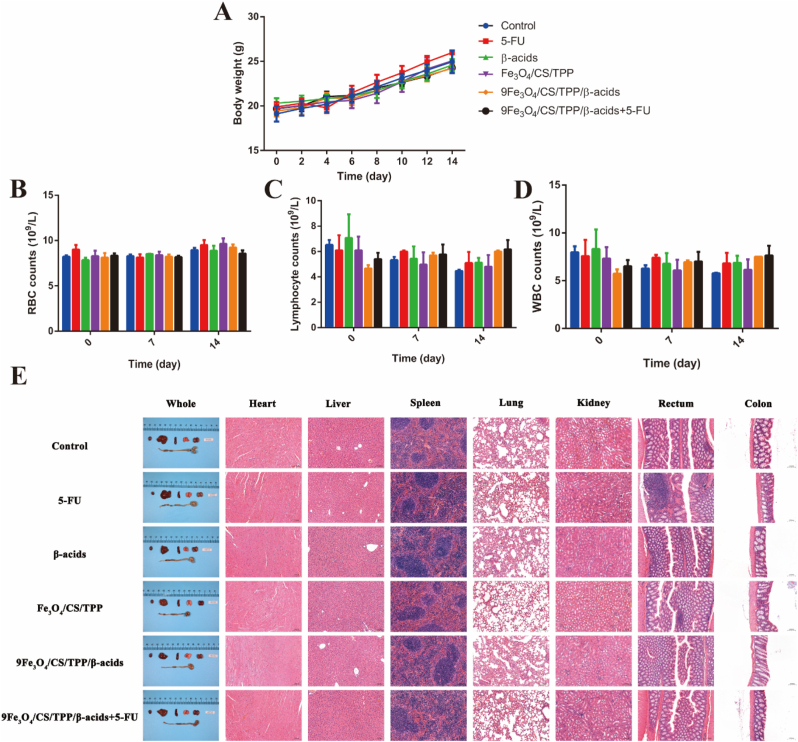
*In vivo* systemic toxicity of nanoparticle. (A) Weight change of healthy C57/BL6 mice (n = 3 mice) treated with PBS, 5-FU, β-acids, Fe_3_O_4_/CS/TPP, 9Fe_3_O_4_/CS/TPP/β-acids and 9Fe_3_O_4_/CS/TPP/β-acids+5-FU, respectively. (B–D) Biochemical analysis of the serum of mice with various treatments (n = 3): (B) red blood cell; (C) lymphocyte; (D) white blood cell. (E) The assessment of main organs by H&E staining (Scale bars are 100 nm).

To assess the safety of drug-loaded nanoparticles in mice, the animals were divided into six groups (Control, 5-FU, β-acids, Fe_3_O_4_/CS/TPP, 9Fe_3_O_4_/CS/TPP/β-acids, and 9Fe_3_O_4_/CS/TPP/β-acids+5-FU), administered and weighed at two-day intervals. Blood samples were collected from mice for routine blood tests on days 0, 7 and 14. The analysis revealed that the drug-loaded nanoparticles exhibited no significant effect on body weight (Fig. 5A), erythrocyte (Fig. 5B), leukocyte (Fig. 5C) and lymphocyte (Fig. 5D) counts. Following drug administration completion, pathological examination of major organs (heart, liver, spleen, lungs, kidneys, rectum, and colon) demonstrated that drug-loaded nanoparticles at 200 mg/kg dosage produced no significant toxic effects on the major organs (Fig. 5E and Fig. S13). These findings confirmed the biological safety of the drug-loaded nanoparticles.

To evaluate the therapeutic efficacy of drug-loaded nanoparticles *in vivo*, this investigation assessed the antitumor effects and safety of 9Fe_3_O_4_/CS/TPP/β-acids and 9Fe_3_O_4_/CS/TPP/β-acids+5-FU by using in situ CRC models. The administration of different drugs occurred through oral or intraperitoneal injection (5-FU) on alternate days. The progression of colorectal cancer was monitored through fluorescence imaging on days 0 and 14, with measurements of tumor weight and body weight recorded. Additionally, H&E staining analysis was performed on colorectal and tumor tissues.

**Fig. 6:**
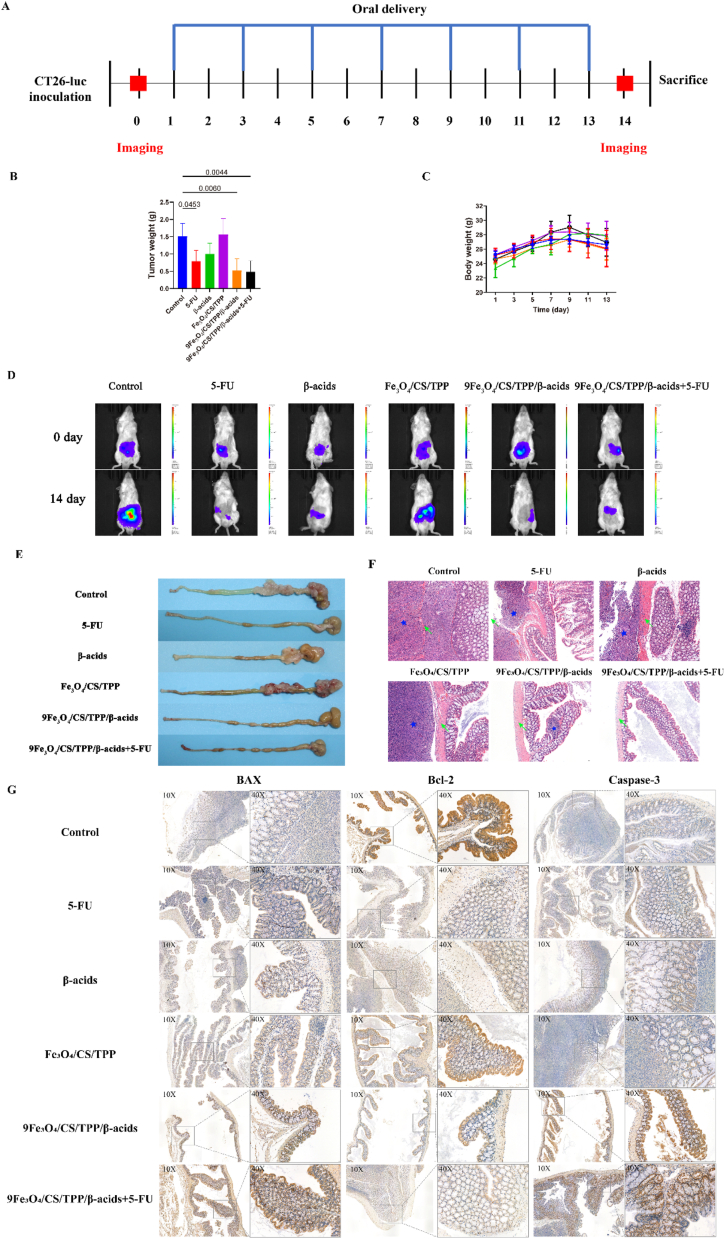
In vivo antitumor activity of 9Fe_3_O_4_/CS/TPP/β-acids. (A) Experimental timeline for the drug treatment groups; (B) Tumor weight of different treatment groups; (C) Body weight of different treatment groups; (D) *In vivo* bioluminescence imaging of CT26 tumor-bearing mice (0^th^ and 14th days). (E) Representative gastrointestinal samples from the indicated treatment groups; (F) H&E staining sections of colorectal and tumor tissue. Blue stars symbolize the tumor regions and green arrows the tumor-invasive colon mucosa. Scale bars are 200 μm. (G) Expression of key apoptosis-related proteins of BAX, Bcl-2 and Caspase-3 in CRC tissues.

*In vivo* therapeutic efficacy of drug-loaded nanoparticles was evaluated through tumor inhibition analysis in mice with in situ colorectal cancer. Following day 5 post-CT26 cancer cell implantation, mice received oral administration of β-acids, Fe_3_O_4_/CS/TPP/β-acids, 9Fe_3_O_4_/CS/TPP/β-acids, and 9Fe_3_O_4_/CS/TPP/β-acids+5-FU on alternate days for 7 consecutive treatments (Fig. 6A). A magnet facilitated nanoparticle removal after each gavage. On day 14, the tumor-bearing mice were euthanized, and tumor dimensions were measured from the extracted colon. Fig. 6B illustrated the *in vivo* antitumor efficacy of drug-loaded nanoparticles in the in situ CRC model, demonstrating reduced tumor dimensions across all experimental groups except the carrier (Fe_3_O_4_/CS/TPP) group relative to controls. Among the experimental groups, the drug-loaded nanoparticle group (9Fe_3_O_4_/CS/TPP/β-acids) exhibited lower CRC tumor weight compared to 5-FU and β-acids groups. The combination of 5-FU with drug-loaded nanoparticles (9Fe_3_O_4_/CS/TPP/β-acids+5-FU) resulted in further tumor weight reduction, suggesting potential synergistic effects. Weight assessment over 14 days revealed an initial increase followed by a decrease, with no significant inter-group variations (Fig. 6C). The antitumor assay safety experiments indicated no significant systemic toxicity from orally administered drugs. Fluorescence imaging results (Fig. 6D) revealed increasing fluorescence intensity in control and vector groups over time post-CT26 cell inoculation. Conversely, treatment groups (5-FU, β-acids, 9Fe_3_O_4_/CS/TPP/β-acids and 9Fe_3_O_4_/CS/TPP/β-acids+5-FU) revealed decreasing fluorescence intensity, indicating tumor inhibition. Notably, 9Fe_3_O_4_/CS/TPP/β-acids and 9Fe_3_O_4_/CS/TPP/β-acids+5-FU treatments resulted in markedly reduced tumor fluorescence intensity. Post-dissection, tumor volume and quantity in treatment groups compared to control and Fe_3_O_4_/CS/TPP/β-acids groups, confirming the drug-loaded nanoparticles' superior tumor growth inhibition capacity (Fig. 6E and Fig. S14). H&E staining (Fig. 6D) revealed typical tumor characteristics in control, 5-FU, β-acids and 9Fe_3_O_4_/CS/TPP/β-acids-treated tissues, including colon wall invasion and mucosal structure disruption. In Fig. 6F, blue asterisks indicate tumor areas, while green arrows denote colonic mucosal tumor invasion. Following 9Fe_3_O_4_/CS/TPP/β-acids treatment, tissue sections exhibited restricted tumor areas and preserved mucosal structures at invasion fronts, indicating therapeutic effectiveness. Combined 5-FU and 9Fe_3_O_4_/CS/TPP/β-acids treatment resulted in intact mucosal structures and near-complete tumor elimination. These findings were compared with previous research (Table S6).

BAX, Bcl-2, and Caspase-3 represent essential regulatory proteins in apoptosis during CRC therapy, with their expression patterns significantly correlating with treatment outcomes, drug resistance, and patient prognosis [[55], [56], [57]]. The present study examined the expression of these apoptosis-related proteins in tumor tissues through immunohistochemical analysis (Fig. 6G and Fig. S15). Through KEGG enrichment analysis, we further clarified that the expression of apoptosis pathway-related genes in the 9Fe_3_O_4_/CS/TPP/β-acids group was significantly enhanced compared with the control group (Fig. S15). The Apoptosis Level of Cells in each group was detected using the Annexin V-7AAD Flow Apoptosis Kit. The results showed that compared with the control group, the apoptosis of HCT116 in the 9Fe_3_O_4_/CS/TPP/β-acids group was significantly increased (Fig. S12). In addition, it has been reported that β-acids may play an antitumor effect by promoting apoptosis of tumor cells, which is consistent with our findings [[58], [59], [60], [61]]. The analysis results demonstrated that both 5-FU and Fe_3_O_4_/CS/TPP/β-acids treatment groups exhibited enhanced expression of BAX and Caspase-3 while showing reduced expression of Bcl-2 compared to the control group, indicating effective activation of apoptotic signaling in colorectal tumor tissues. The combination therapy group (Fe_3_O_4_/CS/TPP/β-acids+5-FU) demonstrated particularly pronounced upregulation of BAX and Caspase-3 expression. These results suggested that the combination therapy effectively enhances apoptotic signaling in tumor cells through synergistic mechanisms, resulting in a more robust antitumor response.

**Fig. 7:**
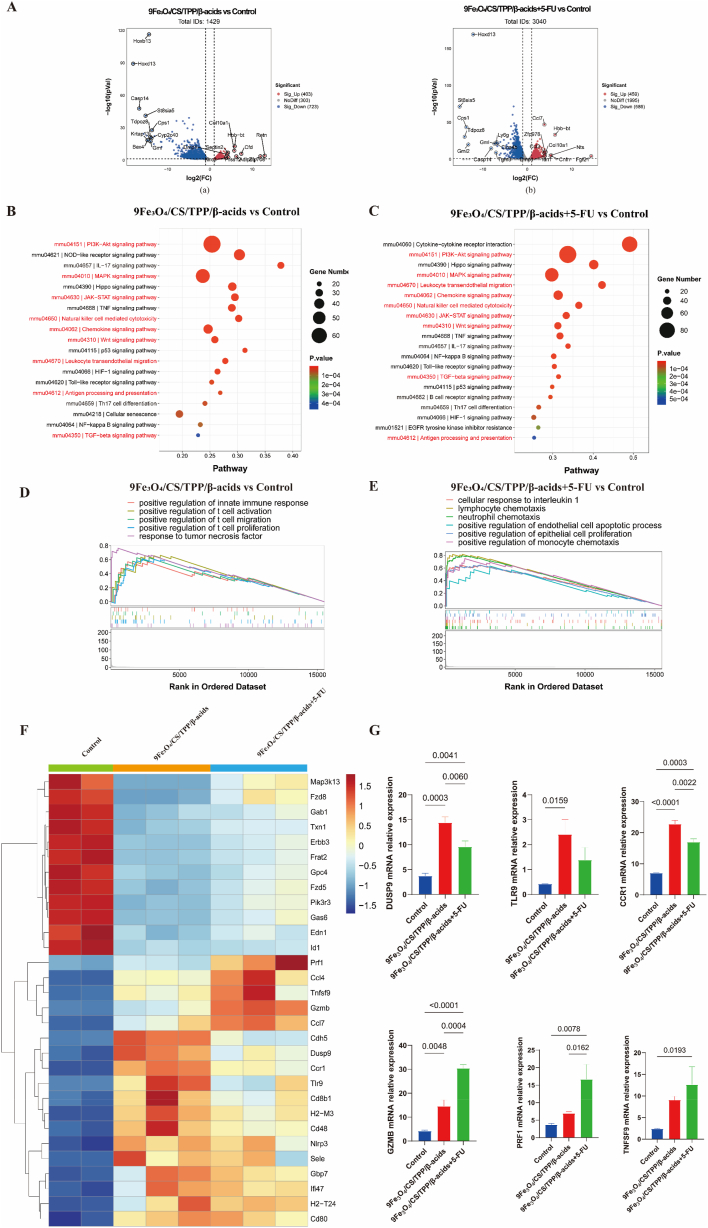
RNA-Seq analysis of 9Fe_3_O_4_/CS/TPP/β-acids, 9Fe_3_O_4_/CS/TPP/β-acids+5-FU treated mice with in situ colorectal cancer. (A) Volcano plots illustrate the DEGs. No differentially expressed genes were denoted as NoDiff. (B) KEGG analysis of DEGs between the Control group and those treated with 9Fe_3_O_4_/CS/TPP/β-acids and (C) with 9Fe_3_O_4_/CS/TPP/β-acids+5-FU. (D) GSEA analysis reveals the pathway enriched in the 9Fe_3_O_4_/CS/TPP/β-acids group and (E) 9Fe_3_O_4_/CS/TPP/β-acids+5-FU group compared to the Control group, respectively. (F) Heatmap of DEGs among the Control, 9Fe_3_O_4_/CS/TPP/β-acids, and 9Fe_3_O_4_/CS/TPP/β-acids+5-FU groups. (G) Quantitative analysis of mRNA expression of DUSP9, TLR9, CCR1, GZMB, PRF1, and TNFSF9 among the Control, 9Fe_3_O_4_/CS/TPP/β-acids, and 9Fe_3_O_4_/CS/TPP/β-acids+5-FU groups.

To elucidate the mechanism underlying the inhibitory effects of 9Fe_3_O_4_/CS/TPP/β-acids and 9Fe_3_O_4_/CS/TPP/β-acids+5-FU on CRC, genome-wide RNA-Seq analysis was performed on in situ cancerous intestinal tissue samples. The study established criteria for screening differentially expressed genes (DEGs) with |log2FC| > 1 and adjusted p-value (padj) < 0.05. The Benjamini-Hochberg (BH) method was applied to control the false discovery rate (FDR) at < 0.05. Based on these criteria, 1126 DEGs were identified between the 9Fe3O4/CS/TPP/β-acids group and the control group, comprising 403 significantly up-regulated and 723 significantly down-regulated genes (Fig. 7A(a)). The 9Fe_3_O_4_/CS/TPP/β-acids+5-FU group exhibited 1045 DEGs compared to the control group, with 459 up-regulated and 586 down-regulated genes (Fig. 7A(b)). KEGG pathway enrichment analysis revealed significant enrichment in key cell death- and tumor-related signaling pathways, including JAK-STAT, PI3K-Akt, ferroptosis, Wnt, p53, MAPK, TNF, cellular senescence, NF-kappa B, necroptosis, and apoptosis. Additionally, immune-related pathways, such as leukocyte transendothelial migration, cytokine−cytokine receptor interaction, B cell receptor signaling pathway, and antigen processing and presentation, showed significant enrichment in both treatment groups (Fig. 7B and C). Gene set enrichment analysis (GSEA) demonstrated upregulation of interferon-related pathways, including cellular response to interferon-β, interferon-γ, and type I interferon signaling in both treatment groups compared to the control group (Fig. S16). These findings indicate that interferon-related pathways, which are integral to tumor immune response, contribute to CRC inhibition in both treatment groups. The 9Fe_3_O_4_/CS/TPP/β-acids group showed significant upregulation of T cell-related pathways involved in tumor cytotoxicity, including positive regulation of T cell proliferation, activation, migration, and innate immune response, along with inflammation-related pathways such as response to tumor necrosis factor (Fig. 7D). The upregulation of these immune-related pathways likely mediates the tumor-inhibitory effects. In the 9Fe_3_O_4_/CS/TPP/β-acids+5-FU group, pathways related to tumor cell apoptosis and epithelial cell differentiation showed upregulation, along with chemokine-mediated pathways including neutrophil chemotaxis, positive regulation of monocyte chemotaxis, lymphocyte chemotaxis, and cellular response to interleukin-1 (Fig. 7E). These alterations may contribute to the enhanced tumor-inhibitory effects observed with the combination treatment. The functional enrichment analysis suggests that 9Fe_3_O_4_/CS/TPP/β-acids primarily exert antitumor effects through immune pathway activation. The addition of 5-FU enhances this effect by simultaneously activating immune responses and directly inhibiting tumor cell proliferation, promoting apoptosis, and resulting in improved tumor inhibition.

Analysis of DEGs between groups demonstrated that, compared to the control group, genes associated with angiogenesis, tumor proliferation, migration, and epithelial-mesenchymal transition (EMT), including EDN1, FZD5, FZD8, GPC4, GAB1, TXN1, FRAT2, GAS6, and ID1, were significantly downregulated in both the 9Fe_3_O_4_/CS/TPP/β-acids and 9Fe_3_O_4_/CS/TPP/β-acids+5-FU groups (Fig. 7F). Additionally, genes involved in the aberrant activation of the PI3K/AKT and MAPK pathways, particularly MAP3K13, ERBB3, and PIK3R3, exhibited significant downregulation (Fig. 7F and Fig. S17). Since aberrant activation of these pathways promotes tumor progression, suppressing these genes may inhibit CRC progression by preventing the abnormal activation of the PI3K/AKT and MAPK pathways. Furthermore, genes associated with T cell activation, natural killer (NK) cell activation, and antigen presentation, including CD80, CD48, H2-M3, and H2-T24, showed significant upregulation (Fig. 7F and Fig. S18), suggesting enhanced immune-mediated tumor suppression. The MAPK pathway negative regulator DUSP9, immune activator TLR9, and chemokine receptor CCR1 demonstrated more pronounced upregulation in the 9Fe_3_O_4_/CS/TPP/β-acids group (Fig. 7F and G), indicating that β-acids-loaded nanoparticles may exert distinct tumor-suppressive effects through MAPK pathway modulation and immune response. Conversely, genes PRF1, TNFSF9, GZMB, CCL7, and CCL4 exhibited more significant upregulation in the 9Fe_3_O_4_/CS/TPP/β-acids+5-FU treated group (Fig. 7F and G and Figure S19). PRF1 and TNFSF9 are linked to the activation of cytotoxic T lymphocytes (CTLs), NK cells, dendritic cells (DCs), and macrophages. The upregulation of effector molecule GZMB directly induces tumor cell apoptosis, inhibits tumor metastasis, and enhances antitumor immune response. The increased expression of chemokines CCL4 and CCL7 is associated with enhanced immune infiltration in the tumor microenvironment, potentially augmenting the antitumor effect. These combined biological functions may explain the stronger antitumor effect observed in the 9Fe_3_O_4_/CS/TPP/β-acids+5-FU group. The transcriptome analysis suggests that both 9Fe_3_O_4_/CS/TPP/β-acids and 9Fe_3_O_4_/CS/TPP/β-acids+5-FU treatments may inhibit CRC progression through the regulation of these molecular targets, suppression of tumor cell proliferation and invasion, and enhancement of immune infiltration within the tumor microenvironment. These mechanisms collectively contribute to CRC inhibition, warranting further investigation into the specific regulatory mechanisms involved in this process.

**Fig. 8:**
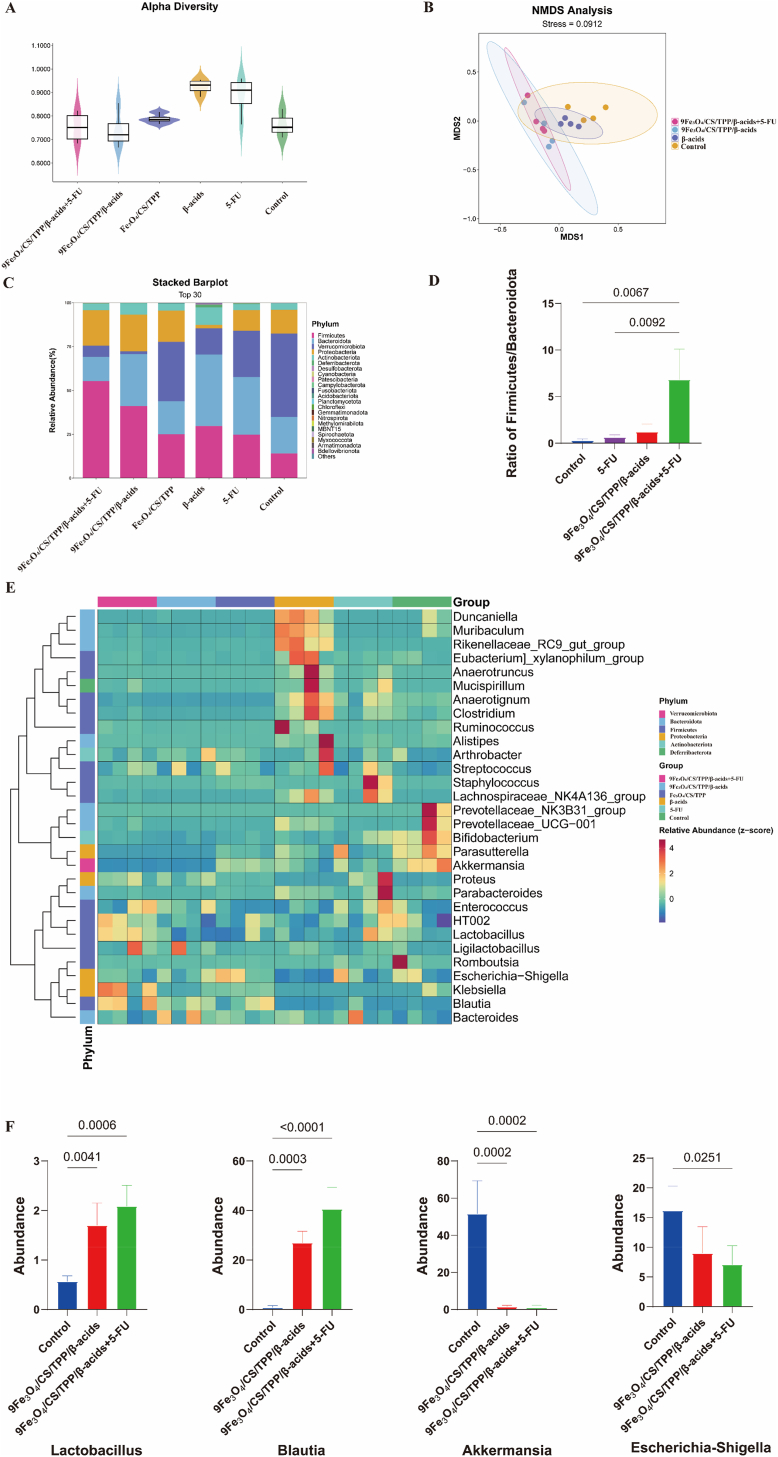
16S rDNA-seq analysis of 9Fe_3_O_4_/CS/TPP/β-acids, 9Fe_3_O_4_/CS/TPP/β-acids+5-FU treated mice with in situ colorectal cancer. (A) Microbial α-diversity in terms of Simpson index at the ASV level; (B) Microbial β-diversity NMDS analysis based on Weighted_Unifrac distance at the ASV level at the end of the treatment; (C) StackedBar plot showing relative abundance of the gut microbiota at the Phylum level; (D) F/B (*Firmicutes*/*Bacteroidota*) ratio among the Control, 9Fe_3_O_4_/CS/TPP/β-acids, and 9Fe_3_O_4_/CS/TPP/β-acids+5-FU groups; (E) Heatmap analysis of the top 30 gut microbiota in different groups at the genus level (displayed as normalized Z-score); (F) Relative abundance of *Lactobacillus, Blautia, Akkermansia* and *Escherichia-Shigella* from different groups.

The composition of gut microbiota revealed a strong correlation with CRC progression [62]. Analysis of the microbial composition by 16S rDNA sequencing evaluated the effects of β-acids and 9Fe_3_O_4_/CS/TPP/β-acids nanoparticles on gut microbiota. Comparison of microbial profiles from mice under different treatments confirmed the modulatory effects of both β-acids and 9Fe_3_O_4_/CS/TPP/β-acids nanoparticles on microbial composition *in vivo*. The 16S rDNA sequencing results revealed no significant alterations in the α-diversity of the gut microbiota (Fig. 8A). However, NMDS analysis (STRESS = 0.0912) demonstrated significant clustering differences in β-diversity for the 9Fe_3_O_4_/CS/TPP/β-acids group compared to the Control and Fe_3_O_4_/CS/TPP group (Fig. 8B). The gut microbiota composition showed marked differences between β-acids direct gavage administration and 9Fe_3_O_4_/CS/TPP/β-acids treatment groups. In contrast, microbial changes in the 9Fe_3_O_4_/CS/TPP/β-acids+5-FU group aligned more closely with the 9Fe_3_O_4_/CS/TPP/β-acids group (Fig. S20). Research indicates that the ratio of *Firmicutes* to *Bacteroidota* (F/B), the two predominant bacterial phyla, exhibits a negative correlation with CRC progression [63,64]. At the phylum level, both 9Fe_3_O_4_/CS/TPP/β-acids and 9Fe_3_O_4_/CS/TPP/β-acids+5-FU groups showed significant increases in *Firmicutes* and decreases in *Bacteroidota* compared to control and 5-FU groups (Fig. 8C). Statistical analysis revealed that while both 5-FU and 9Fe_3_O_4_/CS/TPP/β-acids groups showed an increasing trend in F/B ratio without statistical significance, the 9Fe_3_O_4_/CS/TPP/β-acids+5-FU group demonstrated a significantly higher F/B ratio compared to other groups (Fig. 8D), potentially contributing to the enhanced therapeutic effect observed in the combination treatment group. These findings suggest that 9Fe_3_O_4_/CS/TPP/β-acids+5-FU and 9Fe_3_O_4_/CS/TPP/β-acids nanoparticles may facilitate the rebalancing of gut microbiota composition.

To further examine the effects of 9Fe_3_O_4_/CS/TPP/β-acids on the gut microbiota, the composition of the microbial community was analyzed at the genus level. The analysis revealed that both the 9Fe_3_O_4_/CS/TPP/β-acids+5-FU group and the 9Fe_3_O_4_/CS/TPP/β-acids group substantially increased the abundance of beneficial bacteria, including *Blautia* and *Lactobacillus*, while the relative abundance of the pathogenic bacterium *Escherichia-Shigella* decreased significantly only in the 9Fe_3_O_4_/CS/TPP/β-acids+5-FU group (Fig. 8E). Notably, the abundance of *Akkermansia* significantly reduced in both the 9Fe_3_O_4_/CS/TPP/β-acids+5-FU group and the 9Fe_3_O_4_/CS/TPP/β-acids group compared to the control group (Fig. 8E). Although *Akkermansia* is generally considered beneficial in most studies, recent research suggests it may exacerbate CRC progression under certain conditions [65]. Quantitative analysis demonstrated that the relative abundance of *Blautia* and *Lactobacillus* increased by 30.82- and 46.94-fold, and 2.00- and 2.69-fold, respectively, in the 9Fe_3_O_4_/CS/TPP/β-acids and 9Fe_3_O_4_/CS/TPP/β-acids+5-FU groups compared to the control group. Conversely, the relative abundance of *Escherichia-Shigella* in the 9Fe_3_O_4_/CS/TPP/β-acids+5-FU group was 0.44-fold lower than that in the Control group (Fig. 8F). Furthermore, *Akkermansia* decreased 45.16- and 34.74-fold in the 9Fe_3_O_4_/CS/TPP/β-acids+5-FU and the 9Fe_3_O_4_/CS/TPP/β-acids groups, respectively (Fig. 8F).

These findings indicate that, compared to the 9Fe_3_O_4_/CS/TPP/β-acids group, the combination treatment of 9Fe_3_O_4_/CS/TPP/β-acids+5-FU produced not only a more substantial increase in beneficial bacteria but also demonstrated a more potent inhibitory effect on harmful bacteria. This observation may partially explain, from the perspective of gut microbiota, the enhanced tumor-suppressive impact observed in the combination treatment group on CRC. In conclusion, both 9Fe_3_O_4_/CS/TPP/β-acids+5-FU and 9Fe_3_O_4_/CS/TPP/β-acids treatments effectively enhanced the diversity and abundance of beneficial microorganisms while reducing potentially harmful bacteria, thereby modulating gut microbiota homeostasis and potentially generating synergistic antitumor effects against CRC.

## 4 Conclusion

This study presents the development of an oral therapeutic nanoparticle system for β-acids delivery targeting colorectal cancer. The magnetic chitosan nanoparticles loaded with β-acids exhibited substantial efficacy in inhibiting bacterial proliferation and reducing cancer cell populations *in vitro*. The nanoparticles effectively suppressed tumor growth in a mouse model with no observable adverse effects. Furthermore, this research employed genome-wide RNA-Seq and 16S rDNA-Seq technologies to elucidate the mechanisms through which β-acids-loaded nanoparticles, alone and in combination with 5-FU, inhibit CRC progression at both transcriptional and gut microbiota levels. Future research will focus on establishing a comprehensive dose-response relationship of nanoparticles in mice. The nanoparticles' pH and magnetic responsive behavior, high loading capacity, and sustained-release properties establish as a promising platform for CRC treatment.

## CRediT authorship contribution statement

**Songlin Guo:** Validation, Data curation. **Xia Qiao:** Visualization, Methodology, Data curation. **Lu Ding:** Visualization, Investigation. **Jiayue Liu:** Writing – review & editing, Validation, Supervision. **Yanan Xu:** Data curation. **Jia Cao:** Supervision, Data curation. **Weidong Tian:** Writing – review & editing, Data curation, Conceptualization. **Duan Ma:** Writing – review & editing, Writing – original draft, Data curation, Conceptualization. **Xu Zhang:** Writing – review & editing, Writing – original draft, Resources. **Bingren Tian:** Writing – review & editing, Writing – original draft, Resources, Data curation, Conceptualization.

## Ethics approval and consent to participate

All animal procedures were carried out following the National Research Council's Guide for the Care and Use of Laboratory Animals (8th edition, NIH Publication, 2011). The animal experimental procedures were performed following the regulations of the Medical Ethics Committee of General Hospital of Ningxia Medical University (KYLL-2022-1009 and KYLL-2024-1031).

## Funding

This work was supported by the 10.13039/501100004772Ningxia Natural Science Foundation of China (No. 2023AAC03598 and 2024AAC03598), Special Talent Introduction Project of Ningxia Autonomous Region Key R&D Programs (2023BSB03053 and 2024BEH04151), Yinchuan Science and Technology Plan Project (2024SF044) and Central Guidance Special Fund for Local Scientific and Technological Development of Ningxia (No. 2024FRD05103).

## Declaration of competing interest

The authors declare that they have no known competing financial interests or personal relationships that could have appeared to influence the work reported in this paper.

## Data Availability

Data will be made available on request.
