# Supplementary material for: pH-responsive magnetic Fe3O4 modified chitosan nanoparticles loaded with β-acids to improve colorectal cancer treatment
Source: Mater Today Bio. 2025 Jul 29;34:102151. doi: 10.1016/j.mtbio.2025.102151 (PMC12355082; doi:10.1016/j.mtbio.2025.102151)
Supplement: Multimedia component 1 [file mmc1.docx]

Supporting Information

**pH-responsive magnetic Fe_3_O_4_ modified chitosan nanoparticles loaded with β-acids to improve colorectal cancer treatment**

Songlin Guo ^a, 1^, Xia Qiao ^a, 1^, Lu Ding ^a, 1^, Jiayue Liu ^b^, Yanan Xu ^a^, Jia Cao ^a^, Weidong Tian ^c, *^, Duan Ma ^a, d, *^, Xu Zhang ^a, *^, Bingren Tian ^a, *^

^a^ Surgery Laboratory, Institute of Medical Sciences, General Hospital of Ningxia Medical University, Yinchuan, Ningxia, China

^b^ State Key Laboratory of Quality Research in Chinese Medicine, Institute of Chinese Medical Sciences, University of Macau, Macao, China

^c^ State Key Laboratory of Genetic Engineering and Collaborative Innovation Center for Genetics and Development, School of Life Sciences, Fudan University, Shanghai, China
^d^ Department of Biochemistry and Molecular Biology, Research Center for Birth Defects, Institutes of Biomedical Sciences, Key Laboratory of Metabolism and Molecular Medicine, Ministry of Education, School of Basic Medical Sciences, Fudan University, Shanghai, China

^1^ These authors contributed equally to this work and should be considered co-first authors.

***Corresponding authors:**

**Dr. Bingren Tian,**

Email: tianbingren1@163.com

**Dr. Xu Zhang,**

Email: xuzhang1012@163.com

**Dr. Duan Ma**,

E-mail: duanma@fudan.edu.cn

**Dr: Weidong Tian,**

E-mail: weidong.tian@fudan.edu.cn


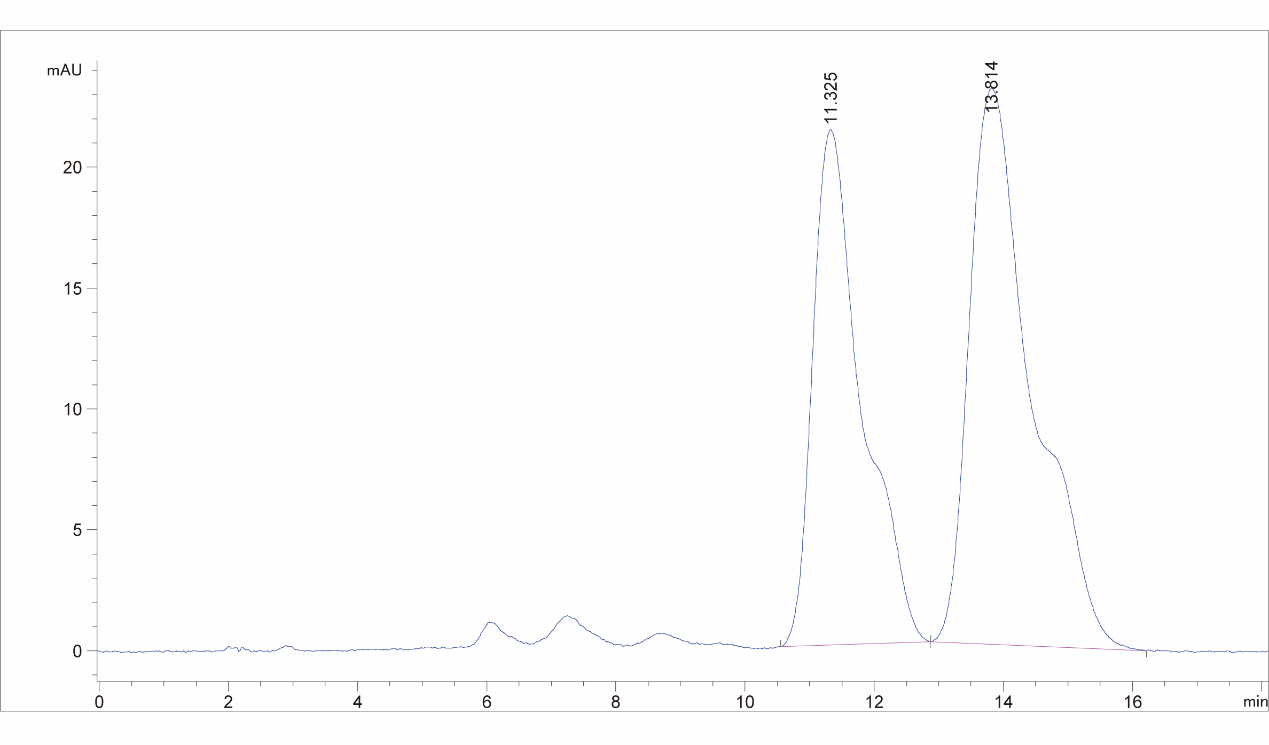


Figure S1. The HPLC of β-acids.


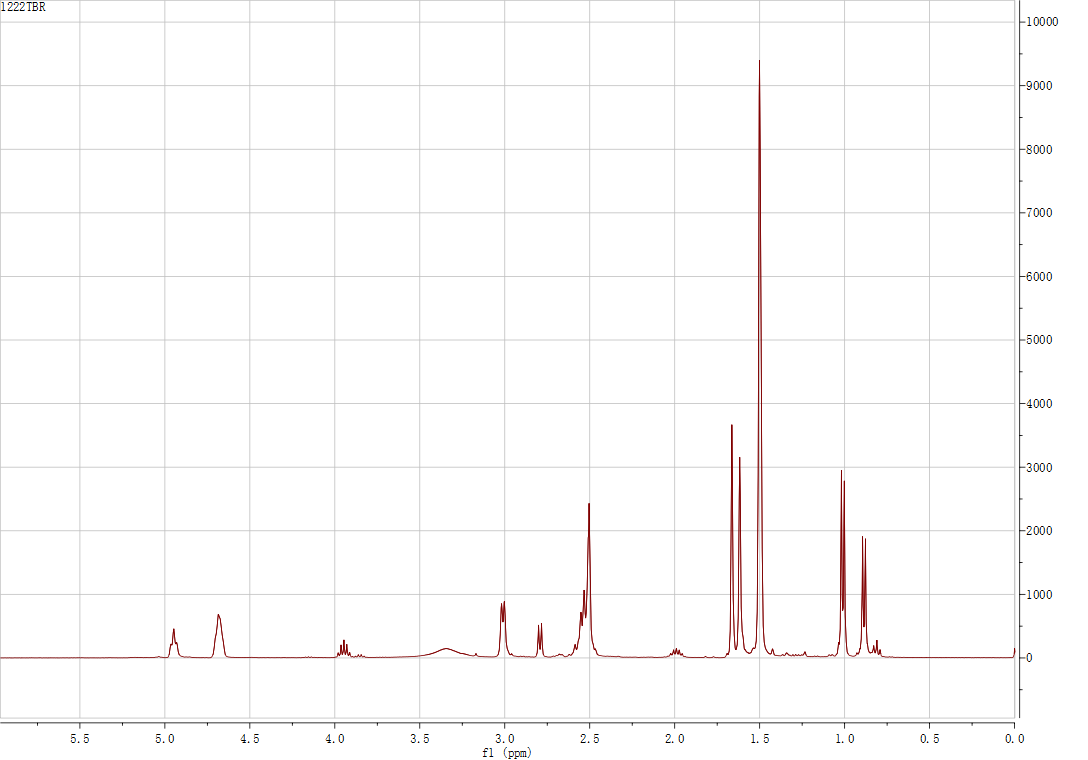


Figure S2. The ^1^HNMR spectra of β-acids.


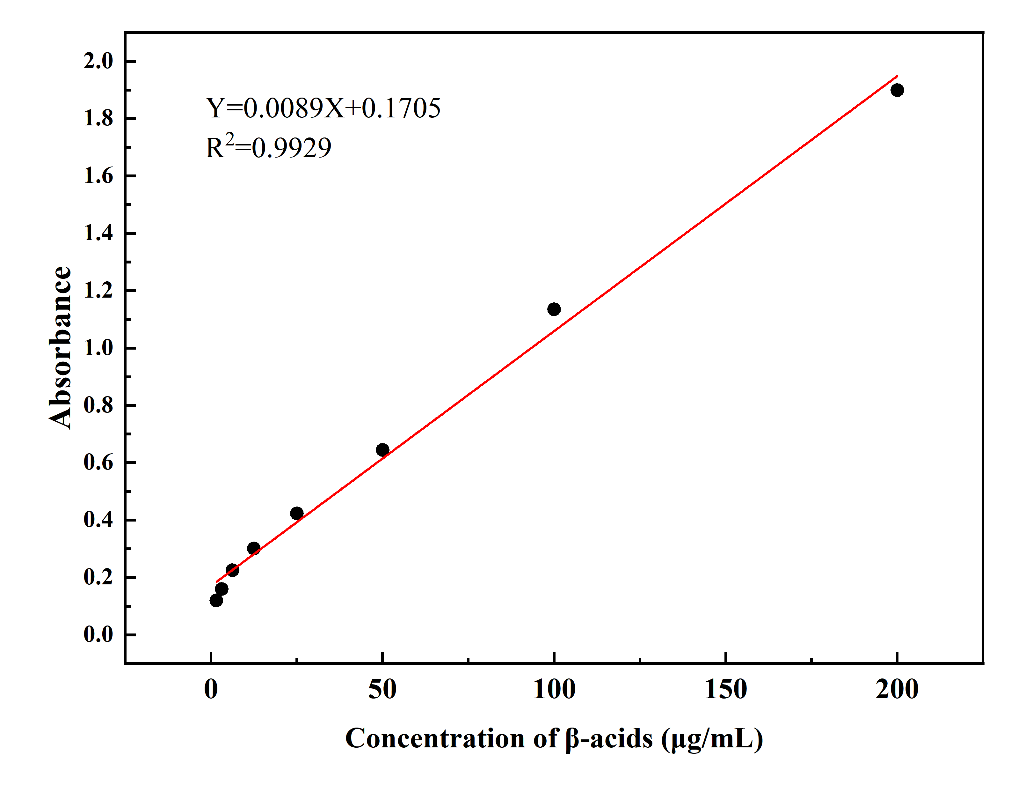


Figure S3. Standard curve of β-acids in ethanol.


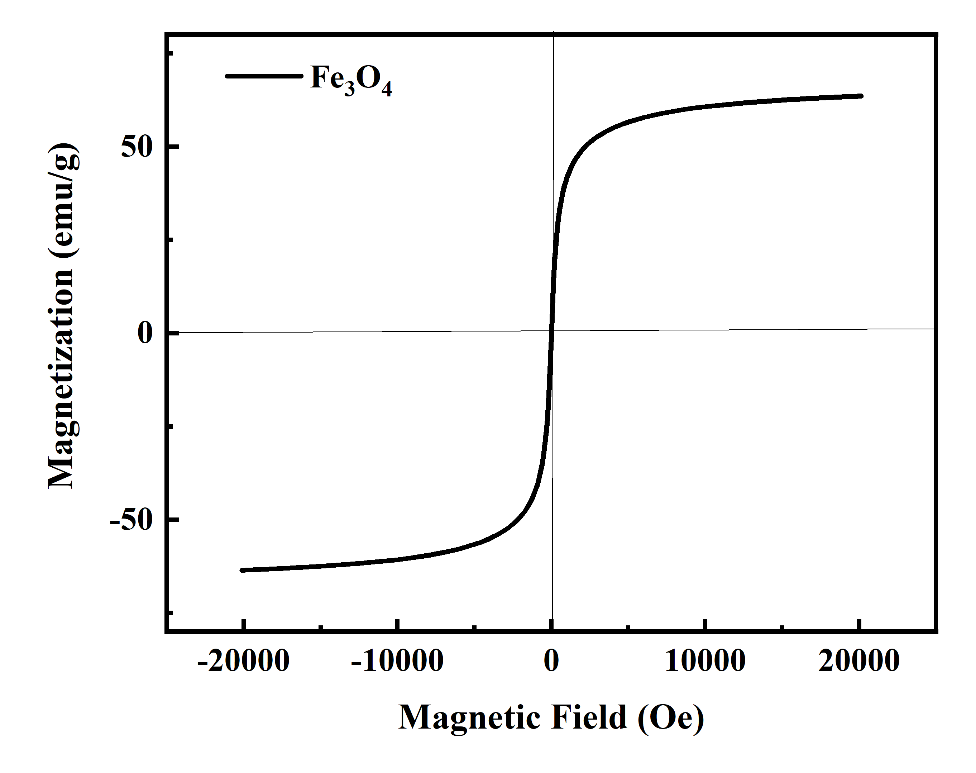


Figure S4. Hysteresis loops of Fe_3_O_4_ measured at 300K.


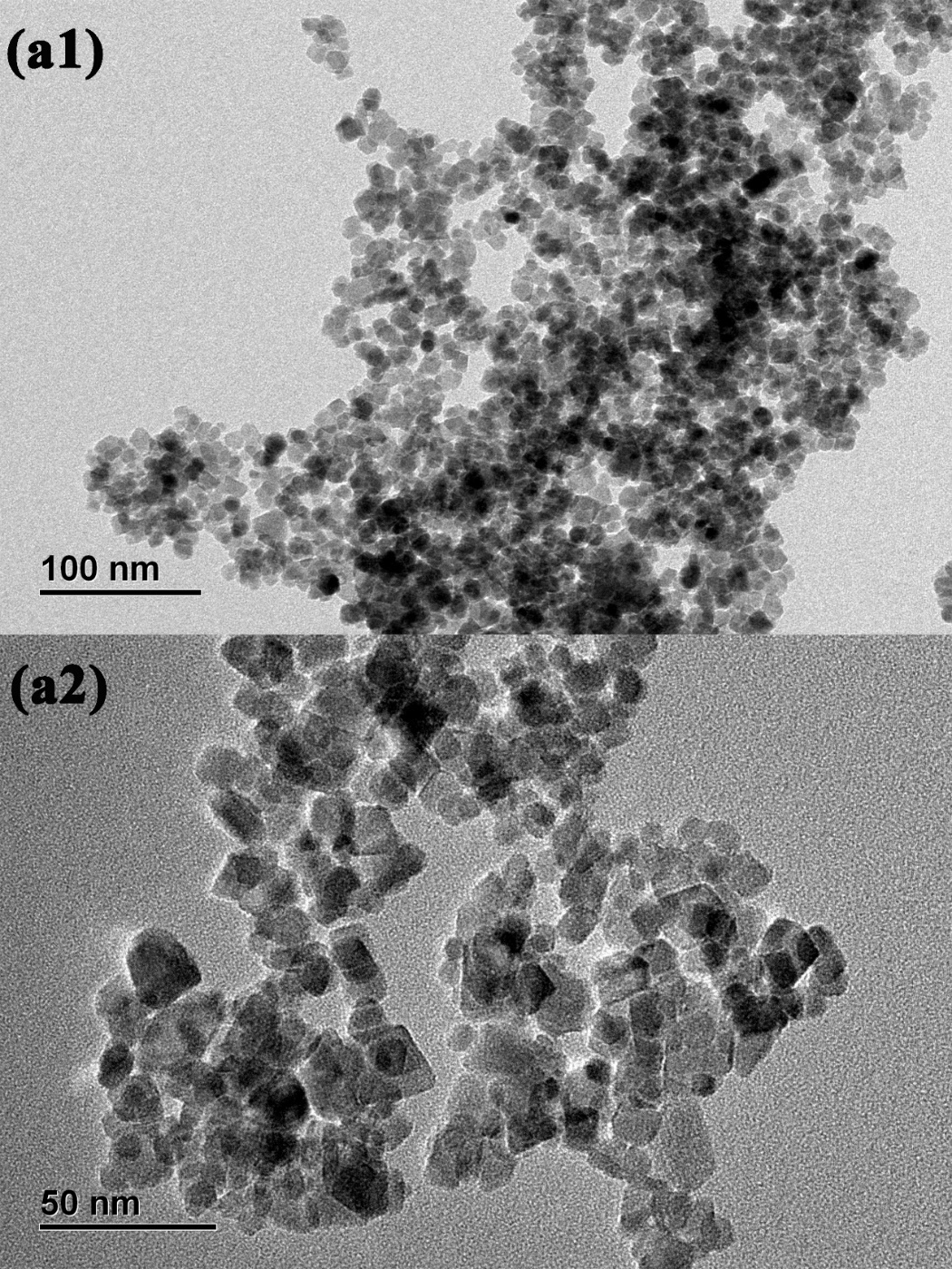


Figure S5. TEM images of Fe_3_O_4_ (a1) 100 nm, (a2) 50 nm.


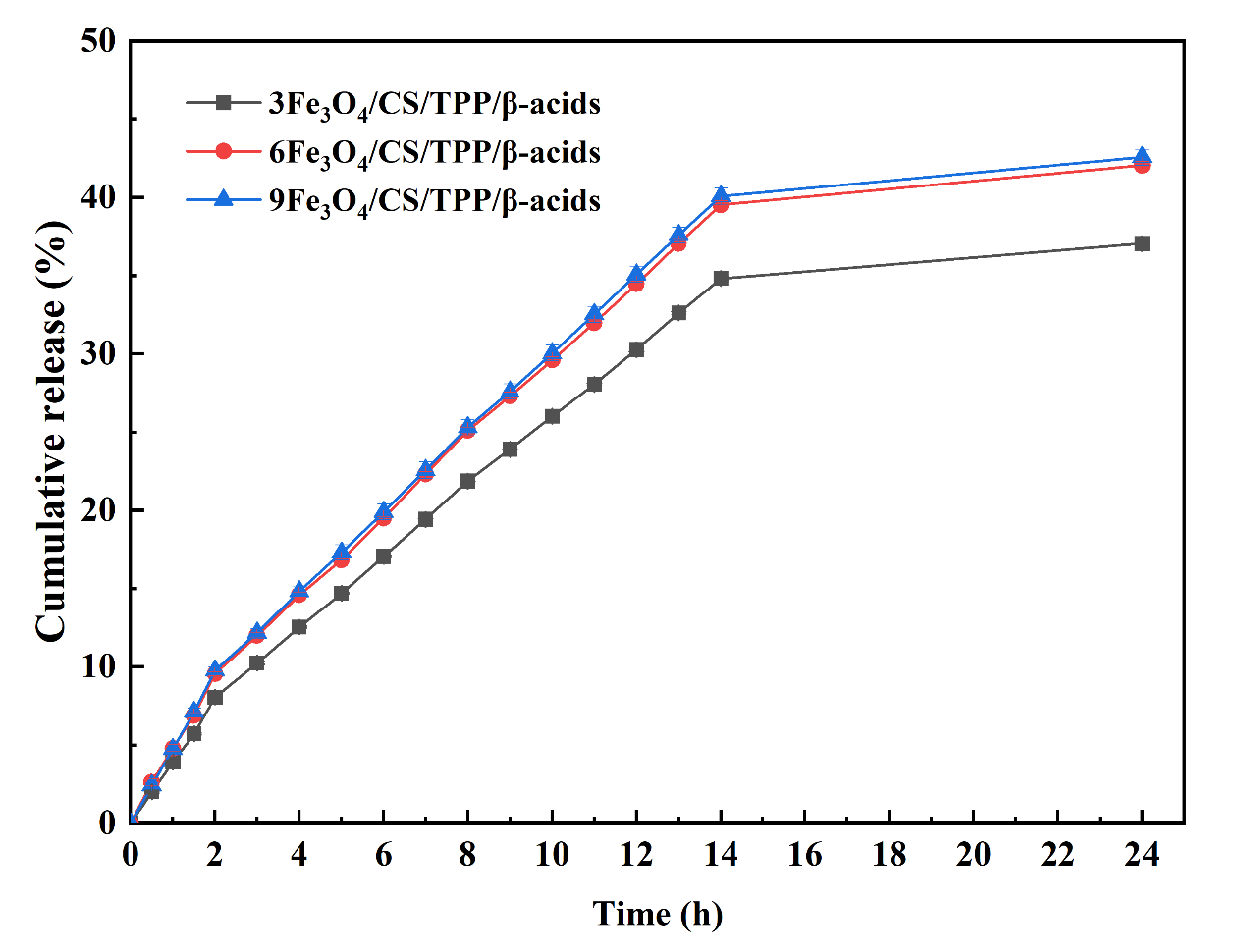


Figure S6. In vitro release profile of β-acids from nanoparticles. For time duration of 0–2 h: Simulated gastric fluid at pH=1.2; For time duration of 2–8 h: Simulated small intestine at pH=6.6; For time duration of 8–24 h: Simulated intestinal fluid at pH=7.4.


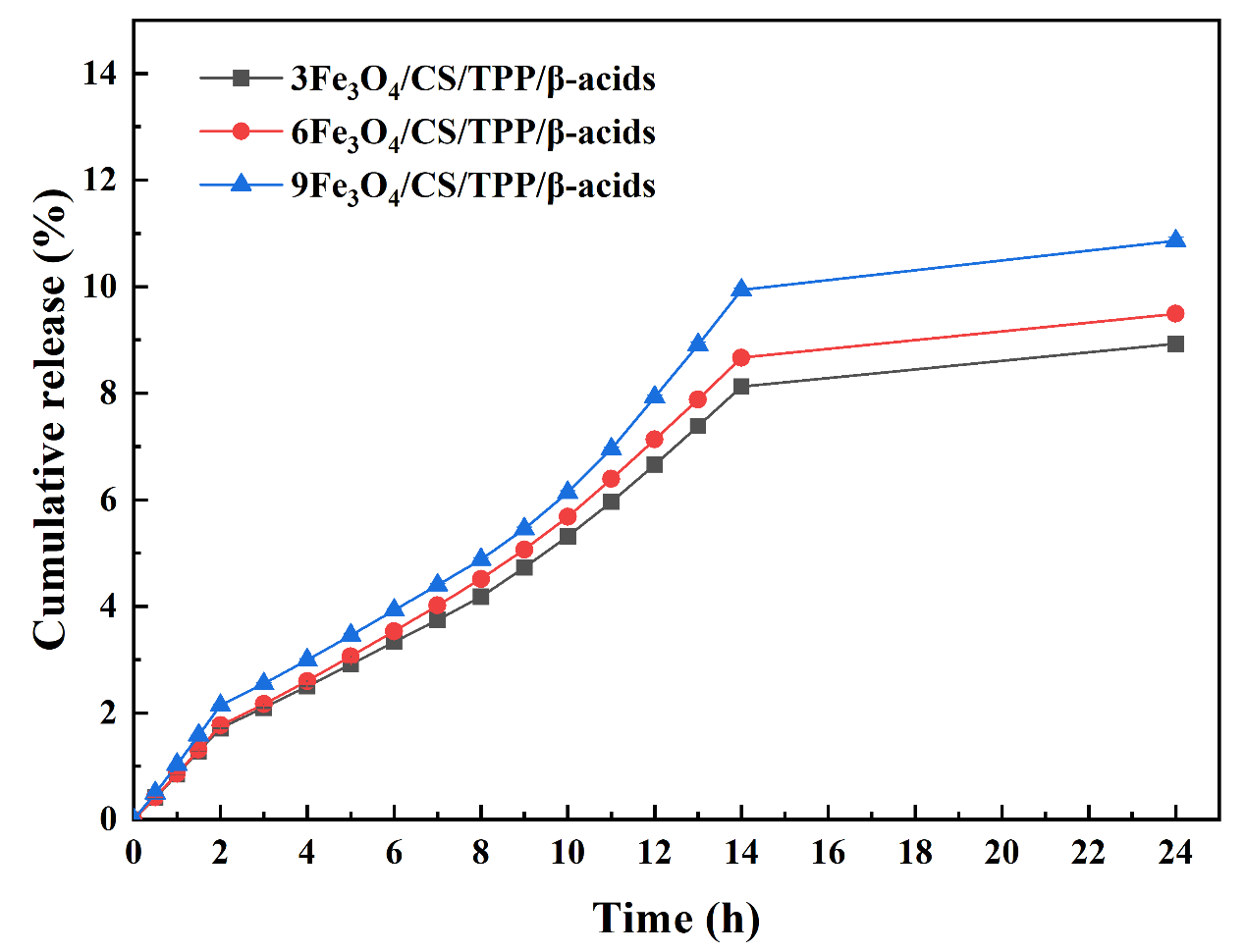


Figure S7. In vitro release profile of β-acids from nanoparticles. For time duration of 0–2 h: Simulated gastric fluid at pH=1.2 with pepsin; For time duration of 2–8 h: Simulated small intestine at pH=6.6 with pancreatin; For time duration of 8–24 h: Simulated intestinal fluid at pH=7.4 with trypsase.


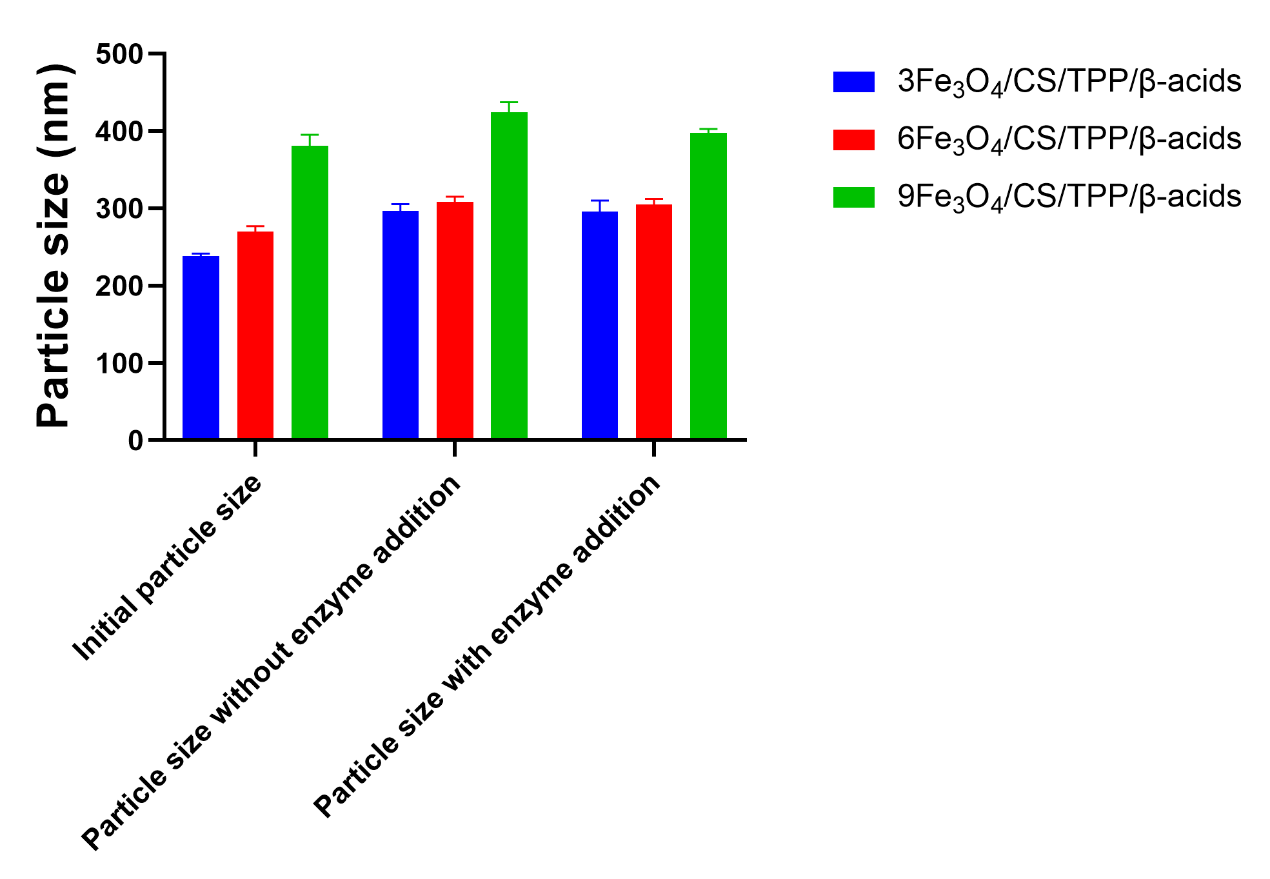


Figure S8. The particle size variations of different nanoparticles under different conditions.


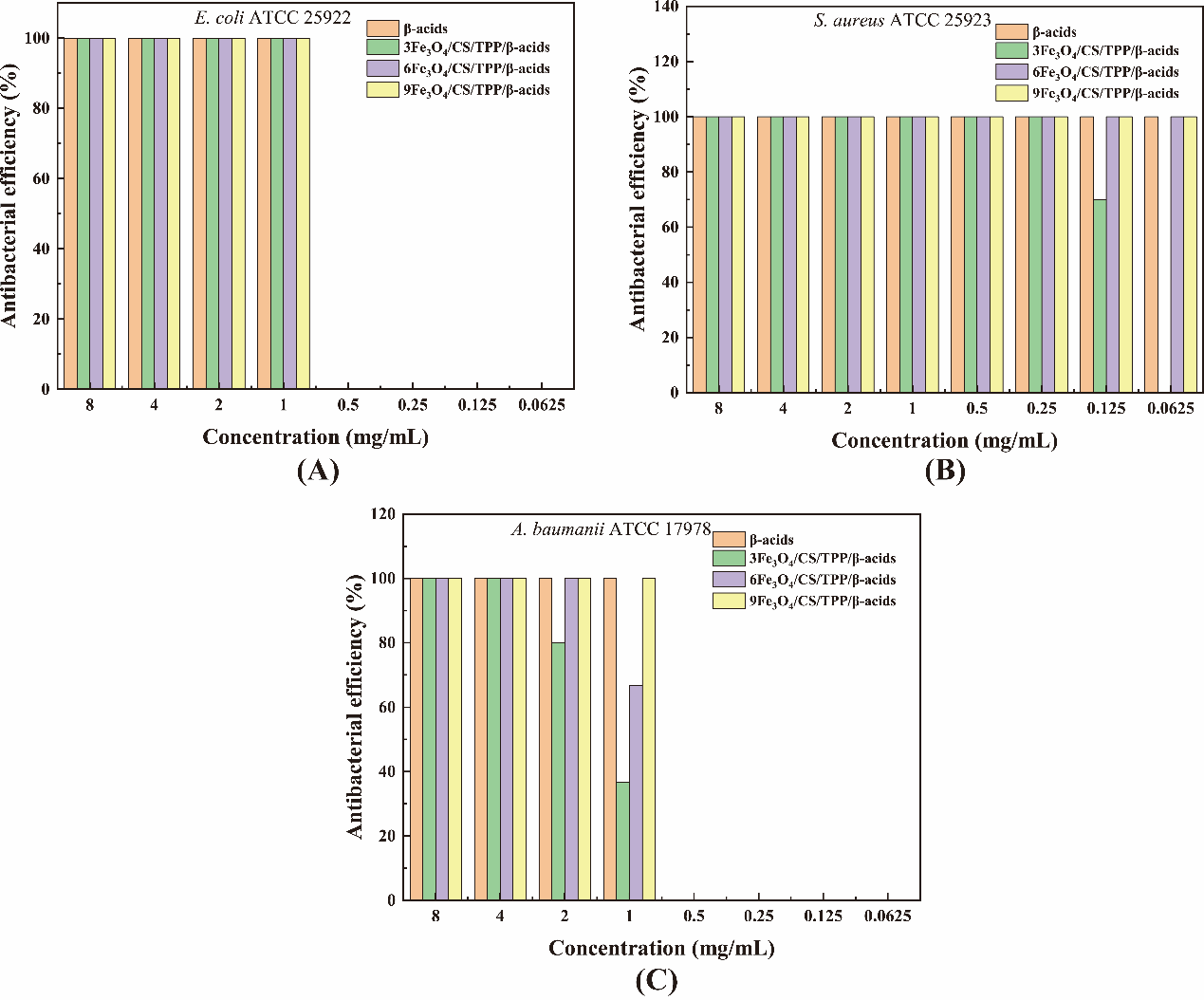


Figure S9. The antibacterial efficiency of nanoparticles against different bacteria.


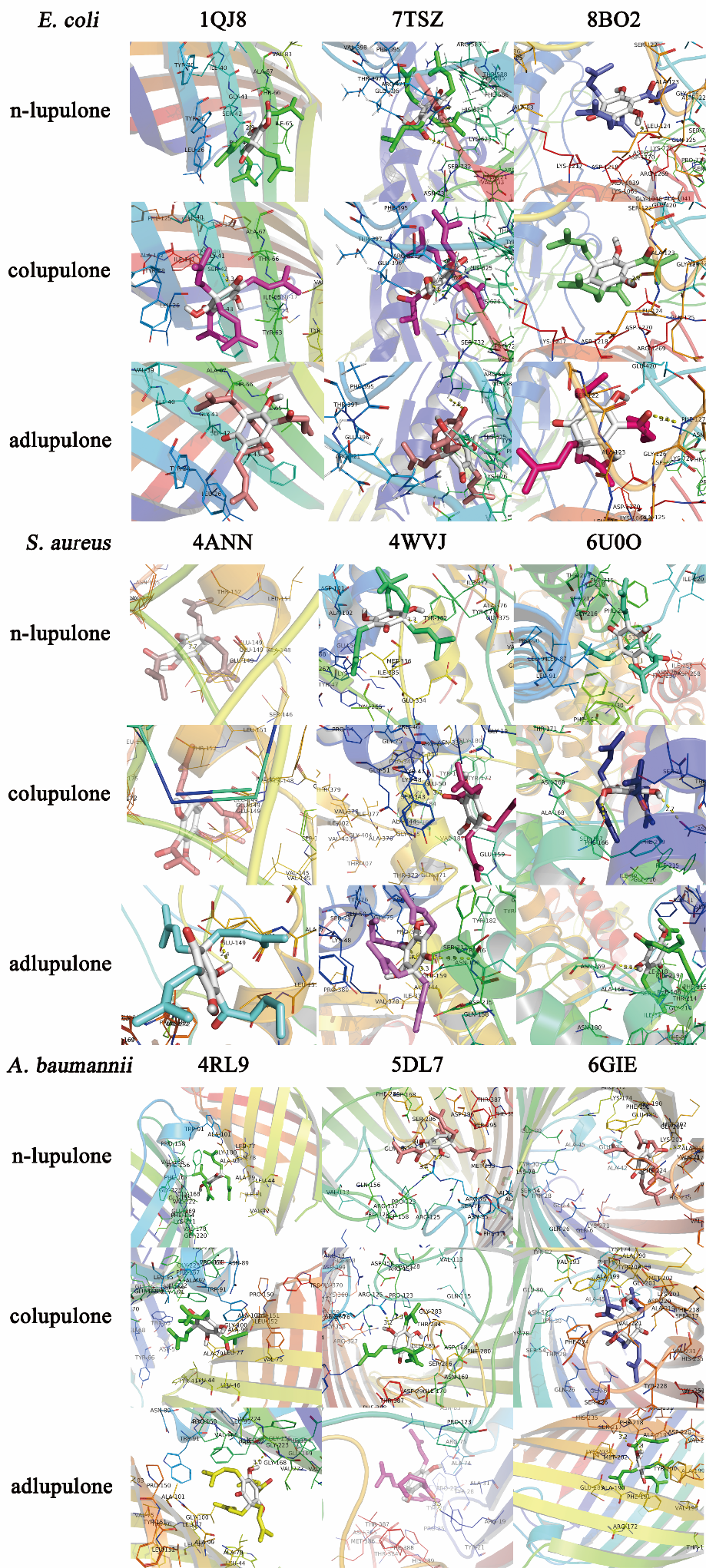


Figure S10. Molecular docking of β-acids with bacteria.


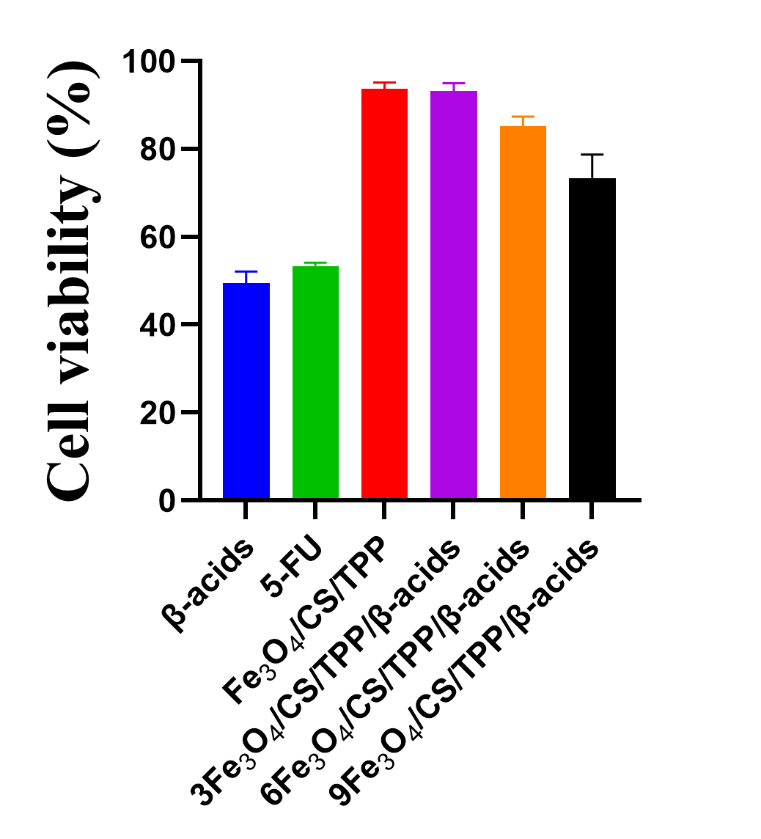


Figure S11. Cell viability of β-acids, 5-FU, Fe_3_O_4_/CS/TPP, 3Fe_3_O_4_/CS/TPP/β-acids, 6Fe_3_O_4_/CS/TPP/β-acids and 9Fe_3_O_4_/CS/TPP/β-acids on CT26. Concentration of nanoparticle is 20 μg/mL.


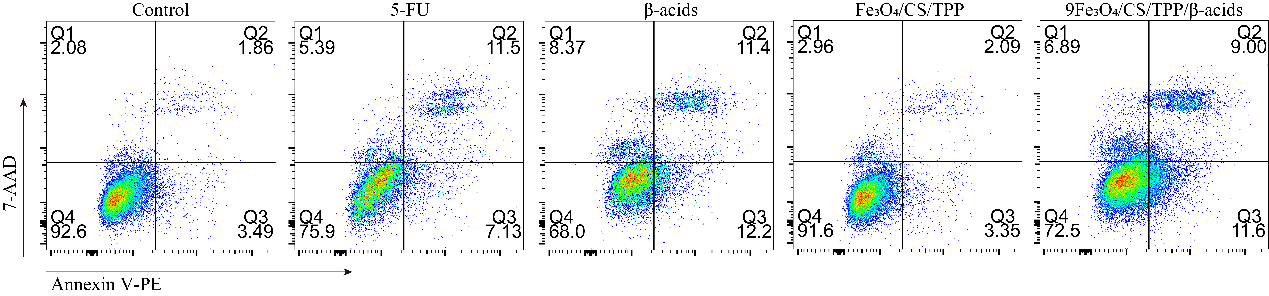


Figure S12. The apoptosis of HCT116 in the 9Fe_3_O_4_/CS/TPP/β-acids.


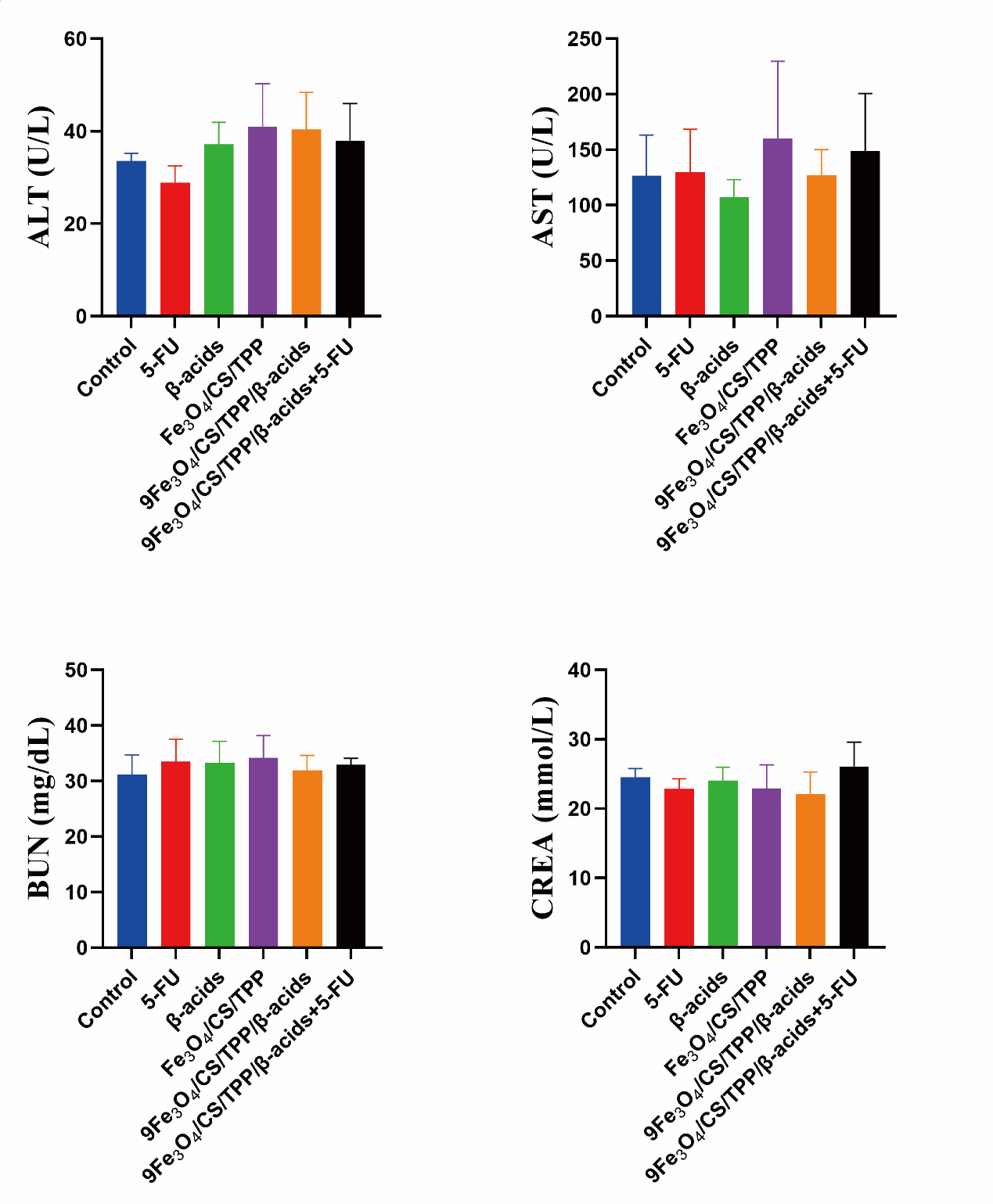


Figure S13. Biochemical analysis of the serum of mice with various treatments: ALT; AST; BUN and CREA (n = 3, time 7 day).


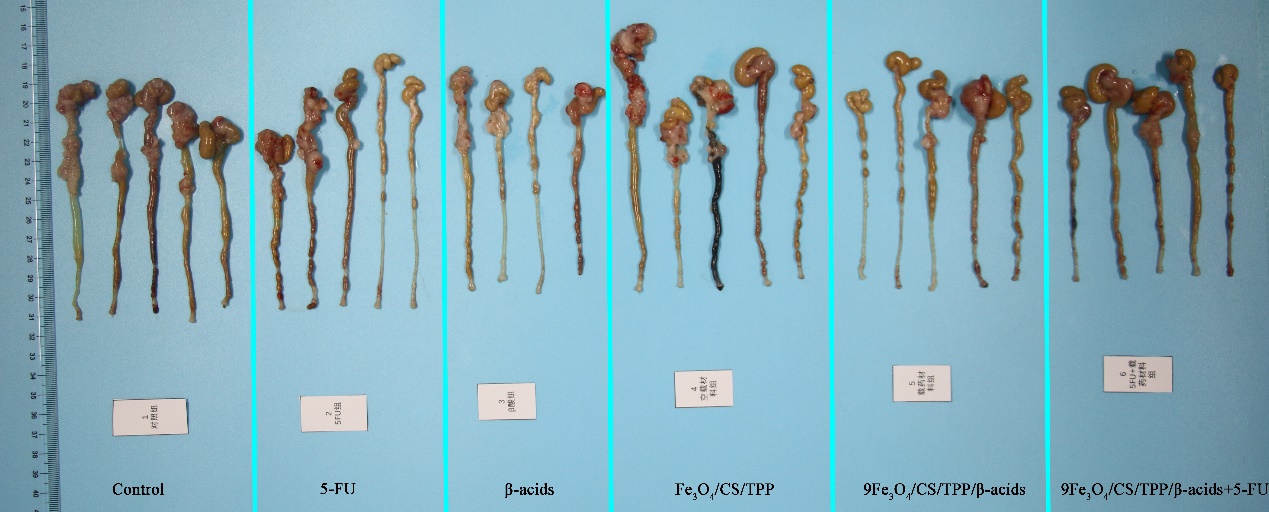


Figure S14. Image of gastrointestinal samples from the different groups.


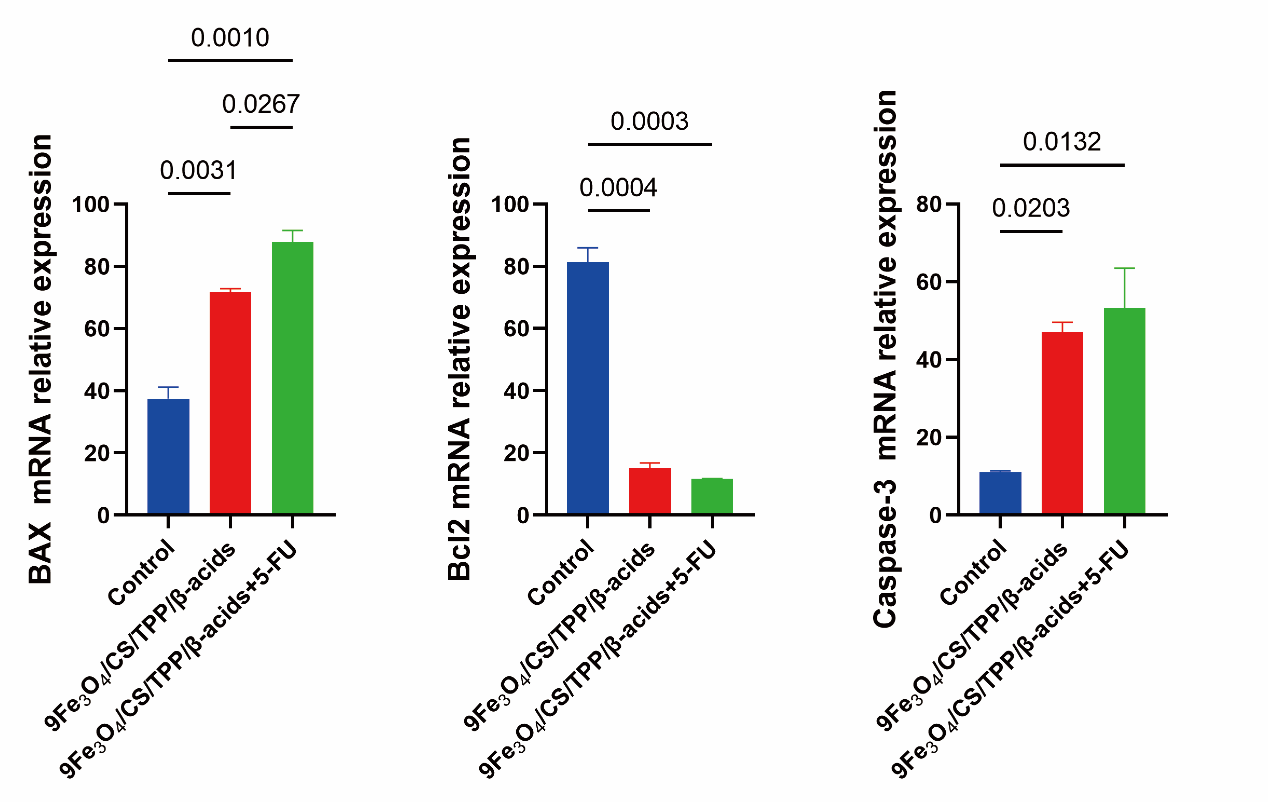


Figure S15. Barplot showing mRNA expression of the differentially expressed genes BAX, Bcl2, and Caspase-3 among the Control, 9Fe_3_O_4_/CS/TPP/β-acids, and 9Fe_3_O_4_/CS/TPP/β-acids+5-FU groups.


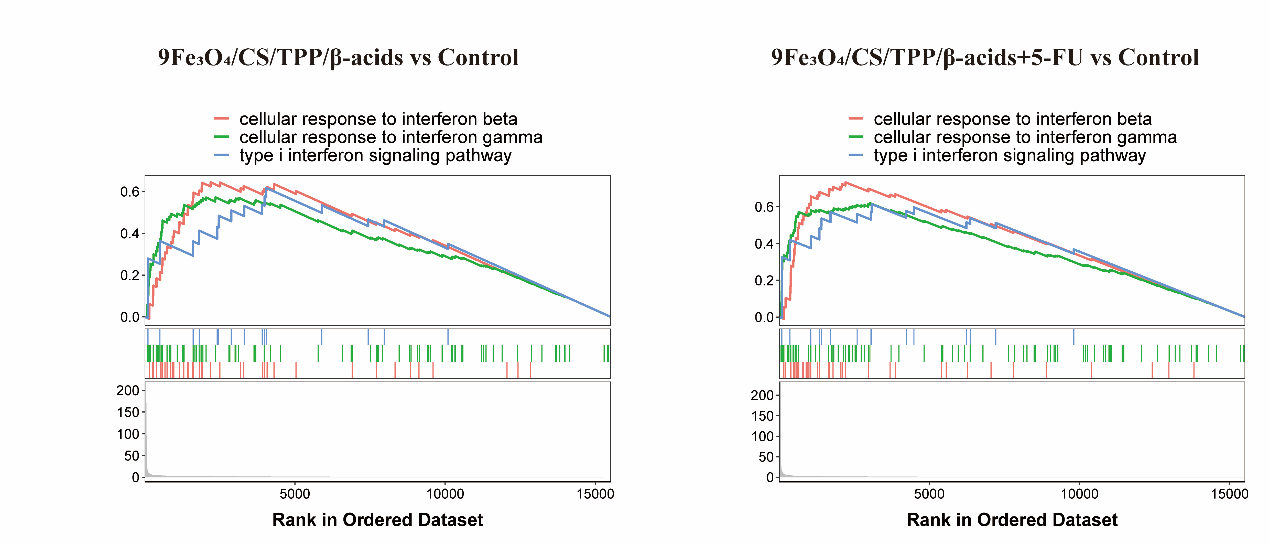


Figure S16. GSEA analysis showed the pathway enriched in 9Fe_3_O_4_/CS/TPP/β-acids group (left) and 9Fe_3_O_4_/CS/TPP/β-acids+5-FU group (right) compared to Control group respectively.


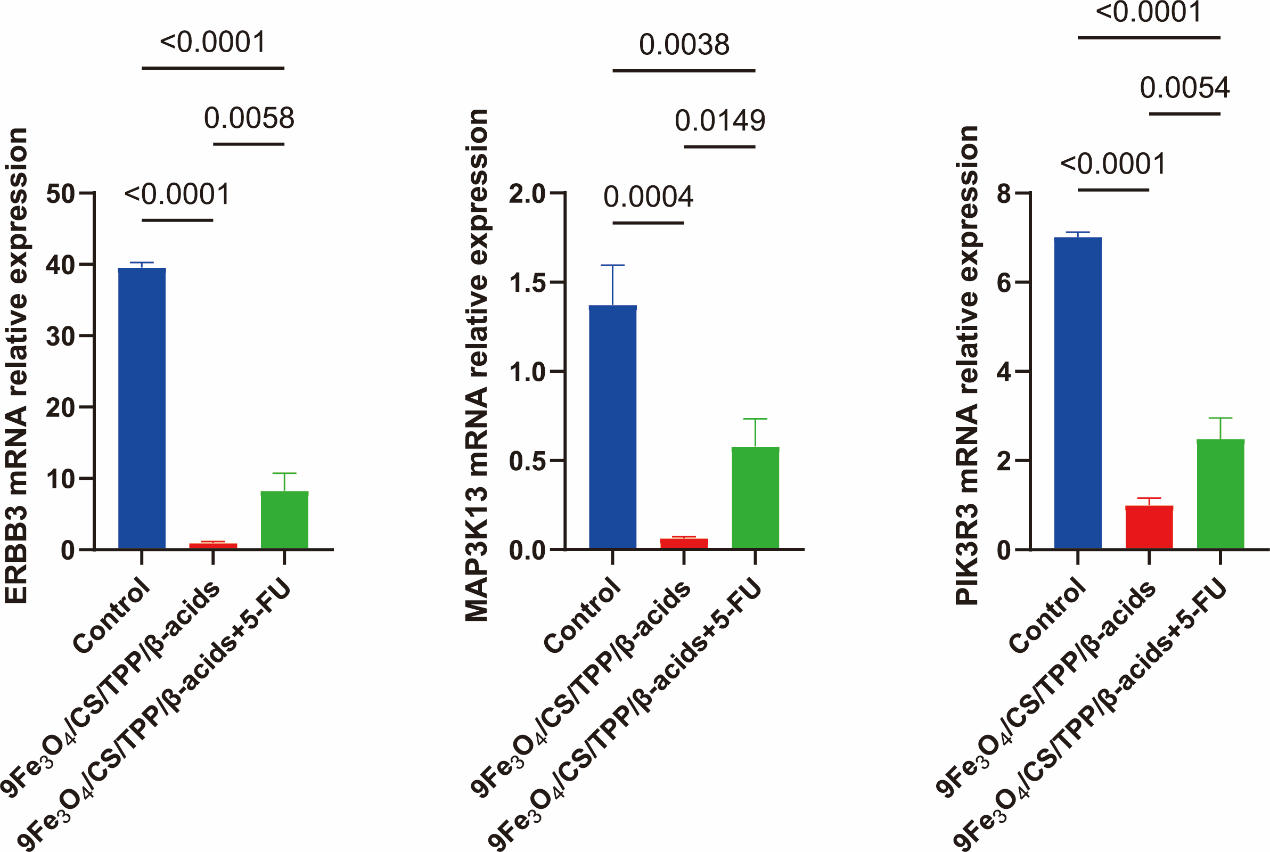


Figure S17. Barplot showing mRNA expression of the differentially expressed genes ERBB3, MAP3K13, and PIK3R3 among the Control, 9Fe_3_O_4_/CS/TPP/β-acids, and 9Fe_3_O_4_/CS/TPP/β-acids+5-FU groups.


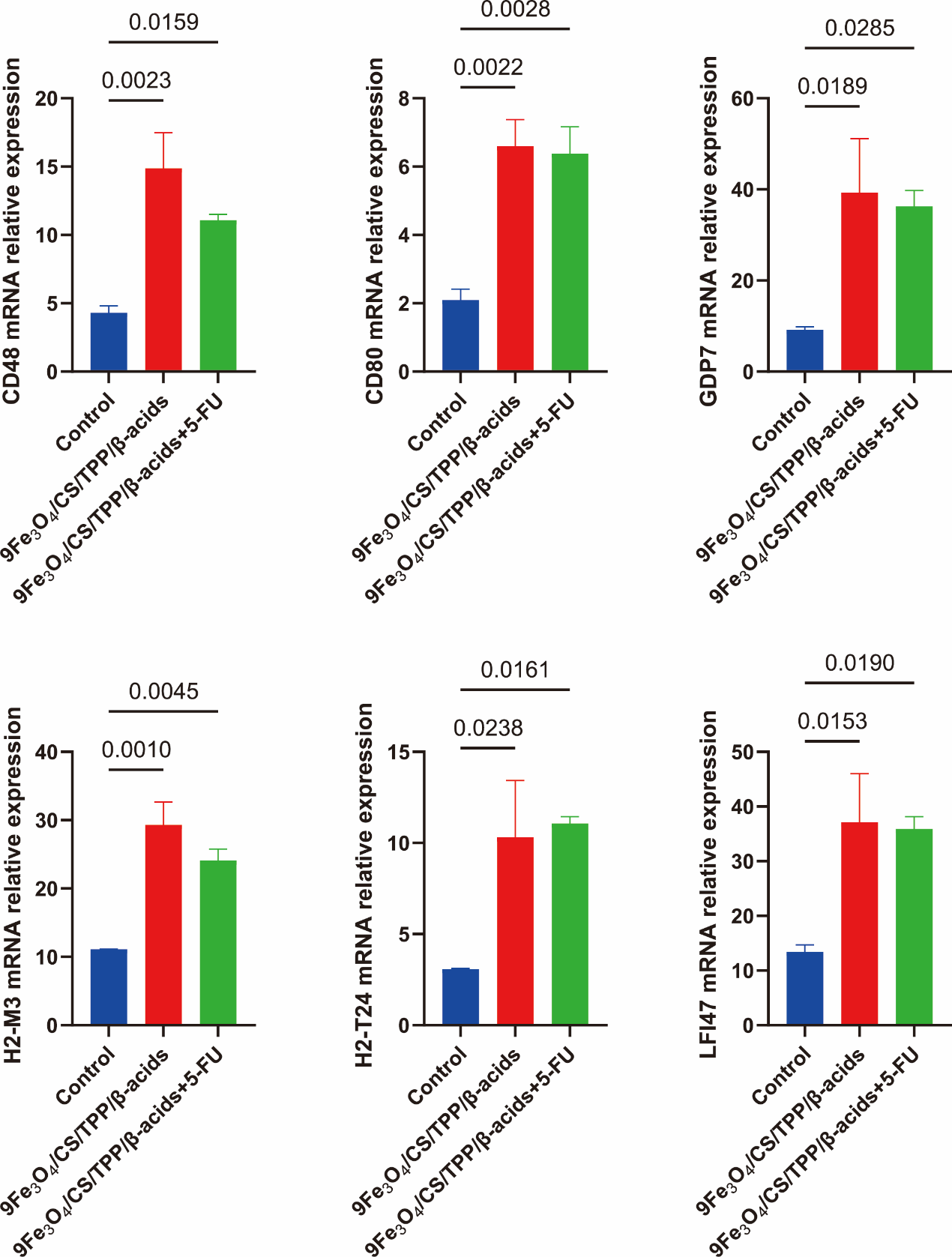


Figure S18. Barplot showing mRNA expression of the differentially expressed genes CD48, CD80, GBP7, H2-M3, H2-T24, and IFI47 among the Control, 9Fe_3_O_4_/CS/TPP/β-acids, and 9Fe_3_O_4_/CS/TPP/β-acids+5-FU groups.


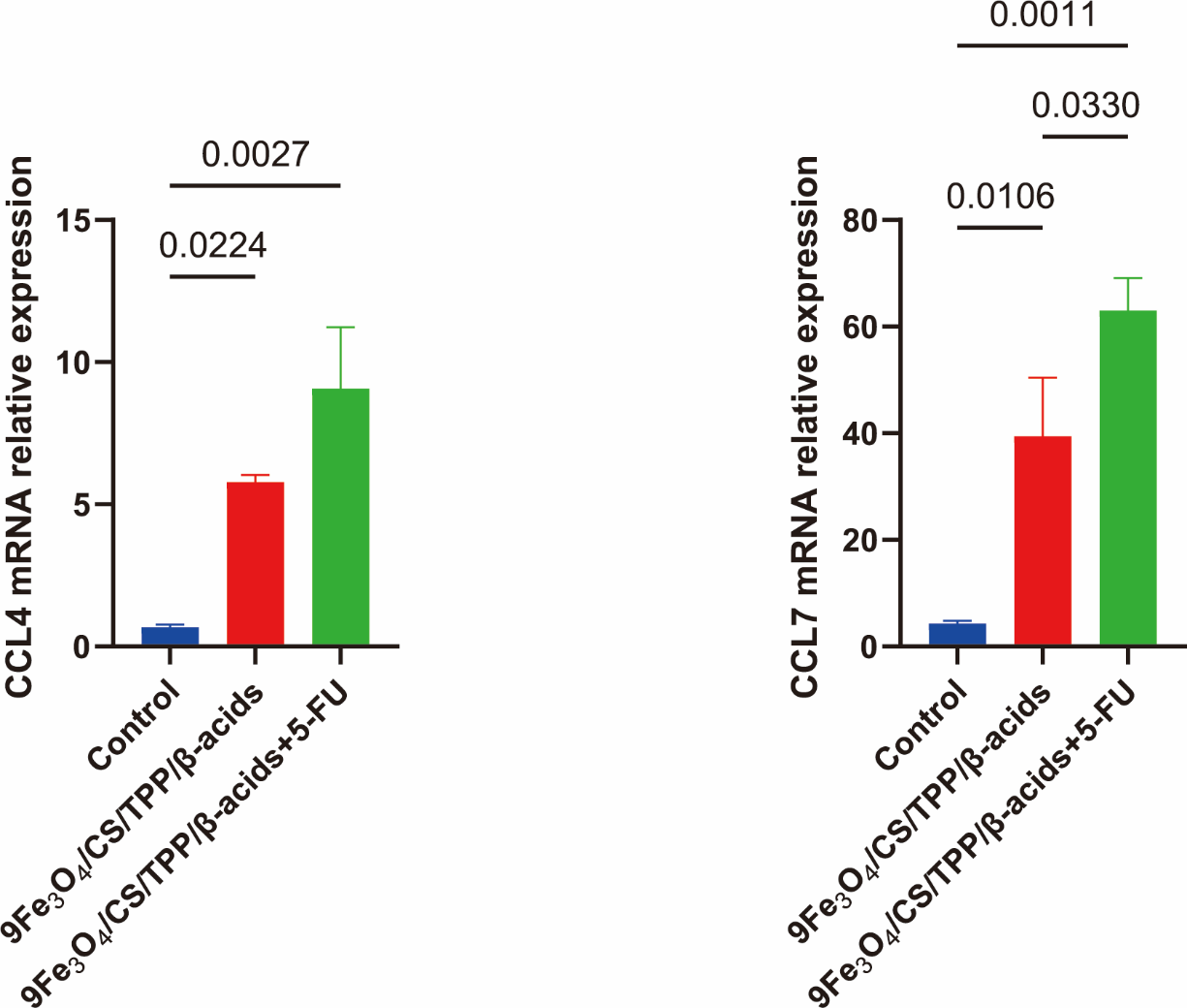


Figure S19. Barplot showing mRNA expression of the differentially expressed genes CCL4, and CCL7 among the Control, 9Fe_3_O_4_/CS/TPP/β-acids, and 9Fe_3_O_4_/CS/TPP/β-acids+5-FU groups.


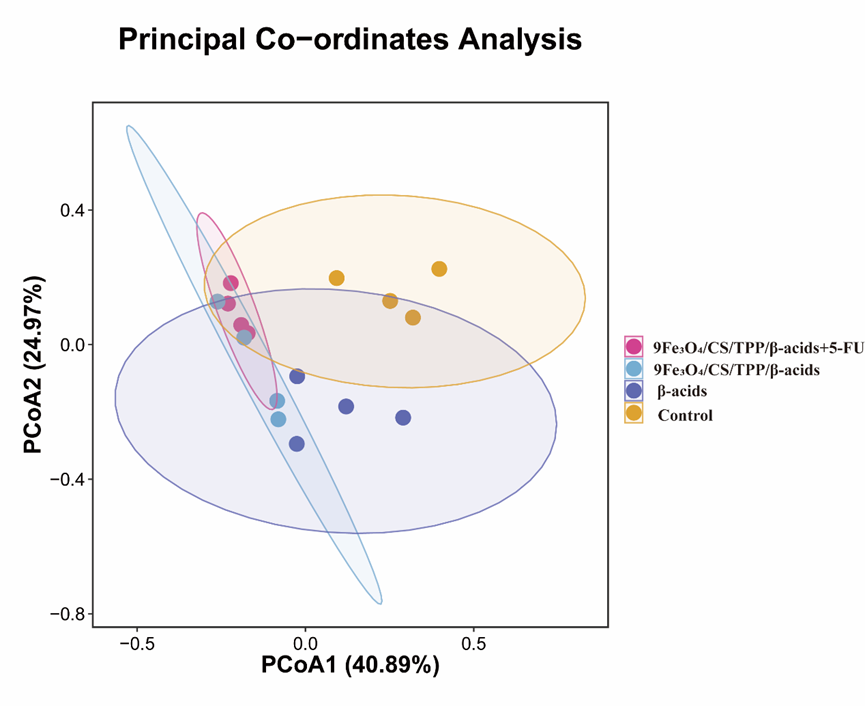


Figure S20. Microbial β-diversity PCoA analysis based on Weighted_Unifrac distance at the ASV level.

Table S1. The composition of nanoparticles.

| Nanoparticle named | Chitosan (CS) (mL) | Sodium tripolyphosphate (TPP) (mL) | Fe_3_O_4_ nanoparticle (mg) | β-acids (mL) |
| --- | --- | --- | --- | --- |
| Fe_3_O_4_/CS/TPP | 200 | 150 | 300 | 0 |
| 3Fe_3_O_4_/CS/TPP/β-acids | 200 | 150 | 300 | 7 |
| 6Fe_3_O_4_/CS/TPP/β-acids | 200 | 150 | 300 | 14 |
| 9Fe_3_O_4_/CS/TPP/β-acids | 200 | 150 | 300 | 22.5 |

Table S2. Parameters of the β-acids release profiles fitting by various models.

| Experiment conditions | Sample | First order | | | Higuchi | | | | Zero order | | | Ritger-peppas | | | |
| --- | --- | --- | --- | --- | --- | --- | --- | --- | --- | --- | --- | --- | --- | --- | --- |
|  |  | k_1_ | a | R^2^ | k_2_ | a | R^2^ | k_3_ | | a | R^2^ | | k_4_ | n | R^2^ |
| pH=7.4 | 3Fe_3_O_4_/CS/TPP/β-acids | 0.032 | 48.18 | 0.6627 | 0.0393 | 34.96 | 0.7142 | 0.0197 | | 34.96 | 0.7142 | | 17.68 | 0.16 | 0.9958 |
|  | 6Fe_3_O_4_/CS/TPP/β-acids | 0.022 | 12.20 | 0.6932 | 0.0107 | 8.31 | 0.6278 | 0.0053 | | 8.31 | 0.6278 | | 3.72 | 0.18 | 0.9845 |
|  | 9Fe_3_O_4_/CS/TPP/β-acids | 0.019 | 14.04 | 0.7588 | 0.0132 | 9.13 | 0.596 | 0.0066 | | 9.13 | 0.596 | | 3.76 | 0.21 | 0.9686 |
| pH=6.6 | 3Fe_3_O_4_/CS/TPP/β-acids | 0.050 | 40.15 | 0.5088 | 0.0295 | 31.28 | 0.8026 | 0.0148 | | 31.28 | 0.8026 | | 18.59 | 0.13 | 0.9713 |
|  | 6Fe_3_O_4_/CS/TPP/β-acids | 0.062 | 6.521 | 0.1945 | 0.0056 | 5.02 | 0.9411 | 0.0028 | | 5.02 | 0.9411 | | 2.97 | 0.13 | 0.9393 |
|  | 9Fe_3_O_4_/CS/TPP/β-acids | 0.035 | 9.481 | 0.7283 | 0.0065 | 7.18 | 0.5563 | 0.0033 | | 7.18 | 0.5563 | | 3.85 | 0.15 | 0.9821 |
| pH=1.2 | 3Fe_3_O_4_/CS/TPP/β-acids | 0.098 | 21.29 | 0.1977 | 0.0104 | 18.59 | 0.8466 | 0.0052 | | 18.59 | 0.8466 | | 13.43 | 0.08 | 0.9934 |
|  | 6Fe_3_O_4_/CS/TPP/β-acids | 0.093 | 5.933 | 0.1344 | 0.0035 | 5.04 | 0.9121 | 0.0018 | | 5.04 | 0.9121 | | 3.47 | 0.09 | 0.9816 |
|  | 9Fe_3_O_4_/CS/TPP/β-acids | 0.065 | 6.688 | 0.3301 | 0.0045 | 5.43 | 0.7966 | 0.0022 | | 5.43 | 0.7966 | | 3.37 | 0.12 | 0.9835 |

Table S3. Determination of total number of bacterial colonies of β-acids loaded nanoparticle against bacteria.

| Sample name | Bacteria | Sample concentration (mg/mL) | | | | | | | |
| --- | --- | --- | --- | --- | --- | --- | --- | --- | --- |
|  |  | 8 | 4 | 2 | 1 | 0.5 | 0.25 | 0.125 | 0.0625 |
| β-acids | S. aureus | 0 | 0 | 0 | 0 | 0 | 0 | 0 | 0 |
|  | E. coli | 0 | 0 | 0 | 0 | >500 | >500 | >500 | >500 |
|  | A. baumannii | 0 | 0 | 0 | 0 | >500 | >500 | >500 | >500 |
| 3Fe_3_O_4_/CS/TPP/β-acids | S. aureus | 0 | 0 | 0 | 0 | 0 | 0 | 10 | >500 |
|  | E. coli | 0 | 0 | 0 | >500 | >500 | >500 | >500 | >500 |
|  | A. baumannii | 0 | 0 | 6 | >100 | >500 | >500 | >500 | >500 |
| 6Fe_3_O_4_/CS/TPP/β-acids | S. aureus | 0 | 0 | 0 | 0 | 0 | 0 | 0 | >500 |
|  | E. coli | 0 | 0 | 0 | 0 | >500 | >500 | >500 | >500 |
|  | A. baumannii | 0 | 0 | 0 | >500 | >500 | >500 | >500 | >500 |
| 9Fe_3_O_4_/CS/TPP/β-acids | S. aureus | 0 | 0 | 0 | 0 | 0 | 0 | 0 | >500 |
|  | E. coli | 0 | 0 | 0 | 1 | >500 | >500 | >500 | >500 |
|  | A. baumannii | 0 | 0 | 0 | 0 | >500 | >500 | >500 | >500 |

Table S4. E_total_ of interaction between β-acids and protein.

| Bacterial | Receptor protein | Type of β-acids | E_total_ (Kcal/mol) | Binding sites | Distance (Å) |
| --- | --- | --- | --- | --- | --- |
| E. coli | 1QJ8 | n-lupulone | -6.7 | SER-42 | 2.9 |
|  |  | colupulone | -6.0 | SER-42 | 3.3 |
|  |  | adlupulone | -5.7 | - | - |
|  | 7TSZ | n-lupulone | -6.7 | ARG-421 | 2.4 |
|  |  |  |  | SER-732 | 2.8 |
|  |  | colupulone | -6.6 | ARG-421 | 2.6 |
|  |  | adlupulone | -6.2 | ARG-583 | 2.8 |
|  | 8BO2 | n-lupulone | -6.2 | LEU-124 | 2.1 |
|  |  | colupulone | -6.4 | ALA-123 | 2.2 |
|  |  | adlupulone | -5.8 | PHE-127 | 3.3 |
| S. aureus | 4ANN | n-lupulone | -6.4 | GLU-149 | 3.7 |
|  |  | colupulone | -6.6 | - | - |
|  |  | adlupulone | -6.4 | GLU-149 | 2.4 |
|  | 4WVJ | n-lupulone | -6.7 | TYR-182 | 3.3 |
|  |  | colupulone | -7.4 | GLU-50 | 3.3 |
|  |  | adlupulone | -6.8 | ASN-156 | 3.5 |
|  |  |  |  | GLU-159 | 3.3 |
|  | 6U0O | n-lupulone | -6.6 | - | - |
|  |  | colupulone | -6.2 | PHE-166 | 2.5 |
|  |  |  |  | ASP-47 | 2.7 |
|  |  | adlupulone | -7.0 | ASN-168 | 3.1 |
| A. baumannii | 4RL9 | n-lupulone | -6.3 | - | - |
|  |  | colupulone | -6.6 | - | - |
|  |  | adlupulone | -6.3 | GLY-168 | 3.0 |
|  | 5DL7 | n-lupulone | -6.9 | ARG-157 | 3.4 |
|  |  |  |  | ARG-75 | 3.2 |
|  |  | colupulone | -6.7 | ARG-125 | 3.2 |
|  |  |  |  | PRO-123 | 3.3 |
|  |  | adlupulone | -5.7 | ARG-19 | 3.5 |
|  | 6GIE | n-lupulone | -5.8 | ALA-219 | 3.2 |
|  |  | colupulone | -6.3 | - | - |
|  |  | adlupulone | -5.6 | SER-217 | 3.2 |
|  |  |  |  | PHE-218 | 2.8 |

Table S5. Selectivity index of nanoparticles.

|  | Selectivity index (SI) | | | |
| --- | --- | --- | --- | --- |
|  | Cytotoxicity (HCT116 and NCM460) | E. coli | S. aureus | A. baumanii |
| 3Fe_3_O_4_/CS/TPP/β-acids | 101.70 and 128.10 | 0.407 and 0.512 | 1.627 and 2.049 | 0.407 and 0.512 |
| 6Fe_3_O_4_/CS/TPP/β-acids | 33.95 and 77.01 | 0.135 and 0.308 | 0.543 and 1.232 | 0.068 and 0.154 |
| 9Fe_3_O_4_/CS/TPP/β-acids | 38.81 and 87.06 | 0.155 and 0.351 | 0.621 and 1.393 | 0.155 and 0.348 |

Table S6. Compare the anticancer efficiency with other literatures

| No. | Drug delivery system | Drug | Animal model | Anticancer efficiency | Reference |
| --- | --- | --- | --- | --- | --- |
| 1 | Albumin nanoparticles functionalized with nucleolin-targeted aptamers | Docetaxel | CT26 mouse model of homogeneous subcutaneous transplantation of CRC | The final tumor volume of the treatment group was 827.19 ± 140.71 mm³, and 2150 ± 270.15 mm³ in the control group. | 10.2147/IJN.S267177 |
| 2 | Metal-organic framework-based nanocarrier with TiO_2_) | TiO₂ | CT26 mouse model of homogeneous subcutaneous transplantation of CRC | The singlet oxygen-consuming nanocarrier nanocarrier treatment group showed the strongest tumor growth inhibitory effect among all treatment groups | 10.1016/j.jconrel.2024.10.020 |
| 3 | Constructed STING agonist-loaded CuS/MnO_2_ bimetallic nanosystem (diAMP-BCM) | BCM | CT26 mouse model of homogeneous subcutaneous transplantation of CRC  MC38 subcutaneous transplanted CRC mouse model | Treatment with diAMP-BCM significantly inhibited tumor growth and tumor regression was observed in some mice. | 10.1186/s12951-024-02970-y |
| 4 | 3,5,4'-trimethoxy-trans-stilbene loaded PEG-PE micelles (BTM@PEG-PE) | 3,5,4'-trimethoxy-trans-stilbene (BTM) | CT26 mouse model of homogeneous subcutaneous transplantation of CRC | Tumor tissue anatomy and weighing showed that the BTM@PEG-PE treatment group had the lightest tumor and the smallest volume; it was significantly better than the free BTM and control group. | 10.2147/ijn.s221625 |
| 5 | Paclitaxel-methylene blue conjugate (PTX-MB) | Paclitaxel-methylene blue conjugate (PTX-MB) | CT26 mouse model of homogeneous subcutaneous transplantation of CRC | The fluorescence intensity of drug-loaded nanoparticles decreased by 44.7 ± 4.8% in the 14th, which had significant antitumor effect compared with the blank PLGA group. | 10.1002/anie.201914120 |
| 6 | Dual-stimulation (pH+magnetic) responsive chitosan nanoparticles (Fe_3_O_4_/CS/TPP) loaded with hops β-acids | β-acids | CRC mouse model of CT26 cell orthotopic transplantation | The tumor weight in each treatment group (except Fe₃O₄/CS/TPP vector group) showed a trend of lower tumor weight compared with the control group. The tumor weight of the 9Fe₃O₄/CS/TPP/β-acids group was significantly lower than that of the β-acids group and the 5-FU single-use group. The tumor weight in the combined drug group (9Fe₃O₄/CS/TPP/β-acids+5-FU) further decreased, suggesting a synergistic anti-tumor effect. | This study |
